# Recursive Editing improves homology-directed repair through retargeting of undesired outcomes

**DOI:** 10.1038/s41467-022-31944-7

**Published:** 2022-08-05

**Authors:** Lukas Möller, Eric J. Aird, Markus S. Schröder, Lena Kobel, Lucas Kissling, Lilly van de Venn, Jacob E. Corn

**Affiliations:** 1 Institute of Molecular Health Sciences, Department of Biology, ETH Zurich, Zurich, Switzerland; 2 Institute of Pharmacology and Toxicology, University of Zurich, Zurich, Switzerland

**Keywords:** CRISPR-Cas9 genome editing, DNA, CRISPR-Cas9 genome editing, DNA damage and repair

## Abstract

CRISPR-Cas induced homology-directed repair (HDR) enables the installation of a broad range of precise genomic modifications from an exogenous donor template. However, applications of HDR in human cells are often hampered by poor efficiency, stemming from a preference for error-prone end joining pathways that yield short insertions and deletions. Here, we describe Recursive Editing, an HDR improvement strategy that selectively retargets undesired indel outcomes to create additional opportunities to produce the desired HDR allele. We introduce a software tool, named REtarget, that enables the rational design of Recursive Editing experiments. Using REtarget-designed guide RNAs in single editing reactions, Recursive Editing can simultaneously boost HDR efficiencies and reduce undesired indels. We also harness REtarget to generate databases for particularly effective Recursive Editing sites across the genome, to endogenously tag proteins, and to target pathogenic mutations. Recursive Editing constitutes an easy-to-use approach without potentially deleterious cell manipulations and little added experimental burden.

## Abstract (web summary)

CRISPR-Cas induced HDR methods tend to have a low efficiency. Here the authors report an HDR improvement strategy, Recursive Editing, that selectively retargets undesired indel outcomes to create additional opportunities for HDR; they introduce REtarget, a tool for Recursive Editing experimental design.

## Introduction

Three major pathways allow cells to resolve DNA double-strand breaks (DSBs) introduced by genome editors such as CRISPR-Cas9^1–3^. Non-homologous end joining (NHEJ) and alternative end joining (Alt-EJ) rejoin DNA ends in an error-prone fashion to produce short insertions and deletions (indels). Homology-directed repair (HDR) utilizes a donor DNA template to repair the DSB in a precise manner^4^. By supplying an exogenous repair template, HDR can be programmed to introduce a versatile spectrum of genomic modifications such as corrections of pathogenic SNPs^5^, precisely targeted deletions^6^, and insertion of large sequence cargoes like chimeric antigen receptors (CARs)^7^. In mammalian cells, HDR frequencies are often low relative to indel formation^3^. This limits its applicability or necessitates subsequent enrichment steps that may be infeasible in therapeutic applications. Thus, developing methodologies to enhance HDR has been an ongoing focus in genome editing.

A range of approaches have been employed to increase HDR frequencies, many of which utilize cellular manipulations to control cell cycle progression^8,9^, inhibit NHEJ pathway mediators^10^, or upregulate HDR-related factors^11^. Other strategies harness Cas9-protein fusions to temporally regulate Cas9 expression^12^, recruit HDR factors^13^, alter the epigenetic state^14^, or physically tether HDR templates to Cas9^15^. Generally speaking, these strategies increase HDR outcomes but can negatively impact genome integrity and cell fitness. They also typically require complex conjugation or precise timing^16^. Consequently, a need exists to further develop HDR enhancement strategies that are simple, safe, and effective.

**Fig. 1:**
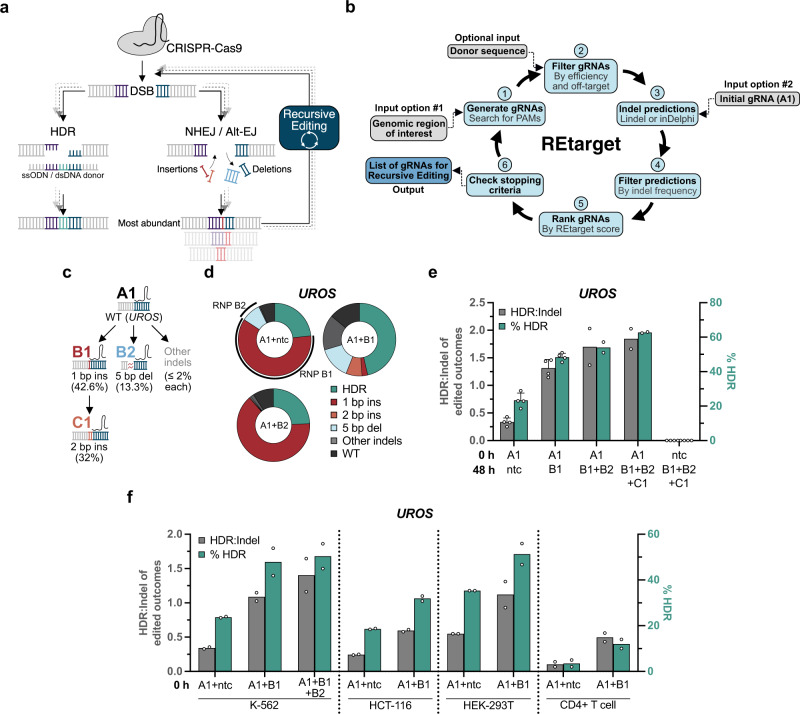
Recursive Editing improves HDR by retargeting indel alleles. **a** A targeted DNA locus is recognized and cut by a Cas9-gRNA complex, creating a DSB. In addition to the desired HDR outcome, undesired indels of various identities are generated via DNA repair pathways like NHEJ and alt-EJ. Retargeting of abundant indels with a new Cas9-gRNA complex yields another opportunity for HDR while simultaneously reducing the number of indel alleles. This recursive strategy can be applied repeatedly. **b** Algorithmic overview of REtarget, a computational tool to find genomic positions amenable for Recursive Editing and generate corresponding gRNAs. **c** Schematic output from REtarget for a given site within the *UROS* gene. gRNA A1 targets the wildtype allele which generates two predominant outcomes, targetable by gRNA B1 or B2. gRNA C1 targets an overall 2 bp insertion created by editing with B1. The listed percentages are the predicted abundance of the given outcome in the corresponding editing event as calculated by Lindel. **d** *UROS*-targeting RNPs complexed with the indicated gRNAs were delivered sequentially in K-562 cells at the indicated time points with an HDR donor that installs a 3 bp insertion. Data are displayed as a percentage of all alleles. **e** HDR:indel ratio on the left y-axis and the corresponding HDR frequency on the right y-axis for sequentially delivered RNPs targeting *UROS* in K-562 cells. **f** Recursive Editing remains effective when all RNPs are simultaneously delivered in a single electroporation (*t* = 0 h) in diverse cell types. Each data point in **d**–**f** represents an individual biological replicate (*n* = 2–4). Error bars + /− standard deviation (SD). Abbreviations: ins insertion, del deletion, ntc nontargeting control, indels insertions and deletions, WT wildtype. Source data are provided as a Source Data file.

Undesired indels are generated when using HDR to make precise genomic modifications^17^. In recent years, several groups showed these NHEJ and alt-EJ editing outcomes behave semideterministically and can be predicted in silico^18–22^. We hypothesized that known or predicted indel identities and frequencies could be utilized to retarget abundant indels for further rounds of genome editing. This would lead to additional opportunities for templated HDR (Fig. 1a). The newly formed HDR product would be additive to the previous HDR outcome and simultaneously decrease the abundance of indels. This approach would not be limited to two cycles but could theoretically be applied recursively if indel outcomes and frequencies allow efficient retargeting. We henceforth refer to this proposed retargeting of undesired editing outcomes as Recursive Editing.

Here, we demonstrate that Recursive Editing is an effective strategy to increase HDR efficiency. We developed a computational tool, termed REtarget, that searches for suitable sites for Recursive Editing at a genomic region of interest and designs the corresponding guide RNAs (gRNAs). Utilizing outputs from REtarget, we demonstrate HDR enhancement by Recursive Editing at numerous sites, in diverse cell types, and with distinct genomic modifications. Through retargeting of non-HDR outcomes, Recursive Editing presents an easy-to-use strategy to boost desired precise genome editing outcomes while simultaneously decreasing unwanted indels.

## Results

### A computational tool to predict suitable gRNAs for Recursive Editing

Recursive Editing depends upon the indel outcomes and frequencies from each round of editing. These could be experimentally determined prior to Recursive Editing, but such an approach would be laborious for even one round of editing and increasingly so as additional rounds are added. We therefore developed a software tool for the design of recursive gRNAs called REtarget (Supplementary Note 1, https://recursive-editing.herokuapp.com). REtarget takes the sequence context around the genomic region or position of interest as input and utilizes Lindel^20^ or inDelphi^22^ to predict indel signatures of individual editing events (Fig. 1b). The first level gRNA can be either designed by REtarget or supplied by the user. The HDR donor sequence can also be provided to ensure it is not targeted. Positions where initial editing is predicted to yield a small number of highly abundant indels are considered for retargeting, serving as entry points for further gRNA sampling. During this subsequent optimization, REtarget samples NGG-PAM motifs proximal to the predicted outcomes of the prior round. REtarget designs the corresponding candidate gRNAs (step 1) and filters out gRNAs with predicted low on-target efficacy and/or high off-target propensity^23,24^ (step 2). For each remaining gRNA, REtarget calculates a score (REtarget score), incorporating both the abundance of the newly targeted indels as well as the fraction of retargetable outcomes (step 3–5). REtarget then selects candidate gRNAs with the highest REtarget scores (step 5) and continues the search recursively. Once the stopping criteria are reached (step 6), REtarget recalculates and selects final level gRNAs based solely on efficacy and specificity. Note that the REtarget score is intended to be used as a priority metric (i.e. comparing potential Recursive Editing reagents at a given locus) rather than as a quantitative predictor of absolute HDR improvement.

In the subsequent sections, we use the following syntax to describe Recursive Editing and its associated gRNAs: each level of editing is denoted as A (first level), B (second level), and C (third level). Each gRNA at a given level is denoted by a number corresponding to the predicted ranked abundance from REtarget. For instance, a gRNA targeting the second most abundant predicted outcome of level two is referred to as gRNA B2 (Fig. 1c). While REtarget can predict editing outcomes using either inDelphi or Lindel, all experiments reported here used Lindel based on our own experience (Supplementary Fig. 1) and previously published comparisons of the two tools^20,25^.

### Recursive Editing converts undesired indels to HDR

We first sought to manually identify a gRNA to test the principle of Recursive Editing, focusing on high overall editing efficiency and formation of one or two primary indels. A previously characterized site within *UROS* fits those guidelines with only two predominant editing outcomes^26^. Using gRNA A1 as an input, we utilized REtarget to predict retargeting gRNAs (Fig. 1c). We then electroporated K-562 cells with a Cas9 ribonucleoprotein (RNP) harboring gRNA A1 in the presence of a single-strand oligodeoxynucleotide (ssODN) HDR donor template encoding a 3 bp insertion. Genome editing was quantified using Illumina amplicon sequencing. We observed an overall editing efficiency of 93.4 ± 2.0 % with a baseline HDR frequency of 23.3 ± 4.2 % (Fig. 1d). A 1 bp insertion accounted for 87.0 ± 0.3 % of all non-HDR outcomes, and a 5 bp deletion was 12.2 ± 0.3 %. Using the in silico predicted gRNA B1 to exclusively target the 1 bp insertion, we delivered RNP B1 and additional ssODN 48 h after RNP A1. After the second round of editing, we observed a near-complete depletion of the 1 bp insertion indel and a commensurate increase in HDR up to 48.4 ± 2.3 %, a 2.1-fold change. The HDR:indel ratio increased from 0.34 to 1.31, meaning the HDR outcome went from being a minority outcome to the majority. Co-addition of RNP B1 with B2 yielded a further increase in HDR. Finally, we added RNP C1 to target the overall 2 bp insertion created by B1. Using all four RNPs in one experiment resulted in an HDR efficiency up to 62.7 ± 0.4%, a factor of 2.7 increase compared to targeting with just RNP A1 (Fig. 1e). Subsequently, the percentage of total indels dropped from 70 ± 3.0 % to 34.3 ± 4.6 %. Recursive Editing depended upon initiation of the editing cascade with RNP A1 since delivery of RNPs B1 B2 C1 alone yielded no detectable editing. Overall, targeting *UROS* illustrates the potential of converting indels to HDR outcomes with Recursive Editing to increase HDR.

Sequential delivery of Recursive Editing reagents is effective but adds additional steps to genome editing workflows. We therefore explored whether simultaneous delivery of multiple RNPs in a single electroporation could drive Recursive Editing. Co-delivery of three RNPs at once (A1 B1 B2) resulted in an improved HDR:indel ratio of 1.4 compared to 0.34 with only RNP A1 (Fig. 1f). The maximal HDR frequency from simultaneous delivery (50.4 ± 7.7 %) was lower than with sequential delivery (62.7 ± 0.4 %). Blocking of the initial target site by co-delivered RNPs could contribute to this effect, even though we did not detect any editing with RNPs B1 B2 C1 (Fig. 1e). Reduced HDR with simultaneously delivered reagents could also be related to the degradation of RNP and/or ssODN over time. However, neither phosphorothioate-protected ssODNs nor multiply-protected synthetic gRNAs yielded a discernible change in editing outcomes (Supplementary Fig. 2). As a precaution, we utilized protected ssODNs for subsequent experiments.

We asked if simultaneous Recursive Editing was effective in multiple cellular contexts by editing *UROS* in HEK-293T, HCT-116, and stimulated human CD4 + T cells. While overall baseline editing and HDR rates differed between cell types, we consistently found HDR to be substantially enhanced and indels decreased when applying Recursive Editing (Fig. 1f, Supplementary Fig. 2). The indel spectra in different cellular contexts were similar, accordant with previously published data^18^. Taken together, simultaneous delivery of Recursive Editing reagents is effective in numerous cell types and significantly simplifies the workflow.

### Genome-wide search for top Recursive Editing-amenable targets

**Fig. 2:**
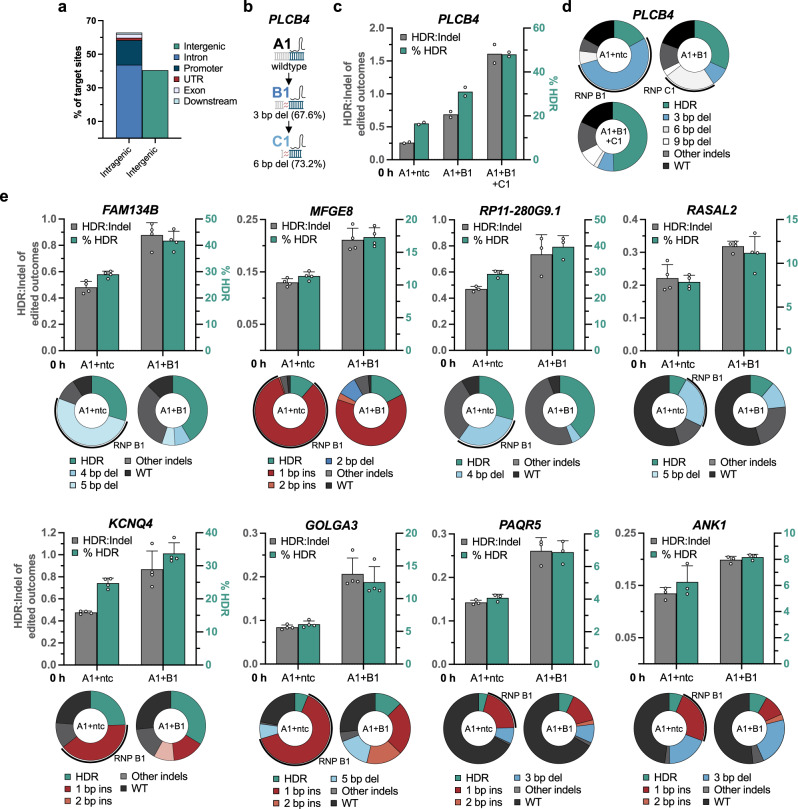
HDR improvement by Recursive Editing is effective at diverse target sites. **a** Genomic location of target sites generated by a genome-wide search with REtarget. Downstream is defined as <3 kb away from a gene. **b** REtarget output for a locus in *PLCB4*. Lindel-predicted abundances for each round of editing are displayed next to the predicted outcome. **c** Recursive Editing at *PLCB4* in HEK-293T cells with the indicated gRNAs, displayed as HDR:indel ratio on the left y-axis and the corresponding HDR frequency on the right y-axis. **d** Individual editing outcomes from **c**. **e** Two-level Recursive Editing outcomes at the indicated locus in HEK-293T cells. The individual editing outcomes are displayed as pie charts below for each locus. The percentage of alleles targeted by RNP B1 are indicated by the outer curve. Each data point represents an individual biological replicate (*n* = 2–4). Axes are scaled for each individual graph. Error bars + /− standard deviation (SD). Abbreviations: ins insertion, del deletion, ntc nontargeting control, indels insertions and deletions, WT wildtype. Source data are provided as a Source Data file.

REtarget can be used to design Recursive Editing reagents for any sequence. Harnessing this capability, we pre-computed Recursive Editing predictions across the entire human genome to evaluate the effectiveness of Recursive Editing at a broad range predicted efficacies and to provide a resource for those wishing to use Recursive Editing. Applying strict search parameters, the genome-wide search resulted in a list of over 23,000 potential Recursive Editing sites (Supplementary Data 1, Supplementary Note 1). The majority of sites (60 %) were within a gene or its 5′/3′ adjacent regions (Fig. 2a).

We targeted twelve unique loci from the pre-computed list with prevalent indels varying from 1 bp insertions to 7 bp deletions using an ssODN encoding a 3 bp insertion. To gain a broader understanding of the requirement of Recursive Editing to improve HDR efficiency, we also assessed loci with suboptimal predicted indel spectra. Targeting *PLCB4* is predicted to generate successive 3 bp alt-EJ deletions in a three-level Recursive Editing system (Fig. 2b). Baseline HDR:indel when editing with RNP A1 alone was 0.26 (16.5 ± 0.7 % HDR) in HEK-293T cells (Fig. 2c, d). Simultaneous editing with RNPs A1 + B1 increased HDR to 30.5 ± 4.0 %. Strikingly, co-addition of RNPs A1 + B1 + C1 resulted in an HDR:indel ratio of 1.61 (48.0 ± 1.4 % HDR). Among eight other loci, two-level Recursive Editing systems led to increased HDR efficiency through retargeting of the primary indel (Fig. 2e). By contrast, in four target sites with inefficient retargeting (*DACT2*, *SLX4*, *LARGE*, and *TEX45*), we observed much lower levels of HDR improvement when adding B-level gRNAs (Supplementary Fig. 3). Overall, REtarget successfully identified genome-wide sites of effective Recursive Editing, and Recursive Editing was an important factor in increasing HDR.

### Recursive Editing enables enhanced installation of large cargoes

**Fig. 3:**
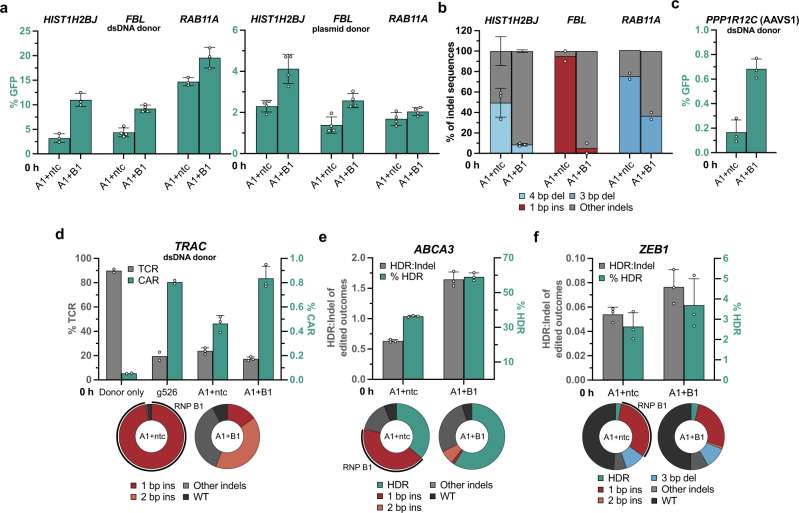
Recursive Editing increases incorporation of large cargo and therapeutically relevant SNPs. **a** Carboxy-terminal GFP tagging efficiency of the indicated genes with Recursive Editing in K-562 cells. **b** Editing outcomes at the indicated target sites without the presence of an HDR donor molecule using Sanger sequencing. **c** Insertion efficiency of GFP at *PPP1R12C* (AAVS1) with Recursive Editing in K-562 cells. **d** Installation efficiency of a chimeric antigen receptor (CAR) at *TRAC* with Recursive Editing in CD4 + T cells. Left y-axis corresponds to the percentage of cells expressing T cell receptor α/β (TCR) and the right y-axis to the percentage of cells expressing the CAR. g526 was a previously identified high performing gRNA^30^. Individual editing outcomes at *TRAC* in CD4 + T cells in the absence of a donor using Sanger sequencing are displayed below. The percentage of alleles targeted by RNP B1 are indicated by the outer curve. **e**, **f** Installation of pathogenic ClinVar variants with Recursive Editing in the listed genes in HEK-293T cells. Individual editing outcomes are displayed below for each locus. Each data point represents an individual biological replicate (*n* = 2–4). Error bars + /− standard deviation (SD). Abbreviations: ins insertion, del deletion, ntc nontargeting control, indels insertions and deletions, WT wildtype. Source data are provided as a Source Data file.

To further investigate the scope of Recursive Editing, we explored whether cargoes larger than the 3 bp insertions tested above could be added via linear dsDNA or plasmid donor templates. We first used REtarget to identify the top Recursive Editing reagents near the start and stop codon of every human gene (Supplementary Note 1). This search yielded 42,787 target sites that fit efficacy and specificity criteria (Supplementary Data 2). We applied this database to select recursive gRNAs to GFP-tag multiple proteins at their carboxy-terminus. Targeting *HIST1H2BJ* with a two-level Recursive Editing system resulted in a substantial increase in HDR in the presence of RNP B1 (2.77 ± 0.53 % to 10.28 ± 0.46 %) using a linear dsDNA donor with 250–350 bp homology arms in K-562 cells (Fig. 3a). Sequencing analysis of the indel profiles in samples without donor present revealed a 4 bp deletion from RNP A1 that was efficiently depleted by RNP B1 (Fig. 3b). An increase in GFP insertion was also observed using a plasmid donor at *HIST1H2BJ*, with overall lower HDR than with a linear donor as previously reported^27^ (Fig. 3a). Recursive Editing enhanced GFP insertion upon co-addition of RNP B1 and efficient retargeting of the primary indel in two other genes, *FBL* and *RAB11A* (Fig. 3a, b). Overall, the database of Recursive Editing reagents targeting human start and stop codons could serve as a resource to facilitate endogenous tagging of proteins.

### Applying Recursive Editing at clinically relevant loci

We subsequently asked if Recursive Editing could be used for sequence modification at clinically relevant sites. Highly effective HDR at the AAVS1 safe harbor locus, located within *PPP1R12C*, could be clinically useful for installing large transgenic cargoes^28^. Using REtarget, we identified a location with high retargeting potential consisting of two initiating gRNAs A1 and A2 (Supplementary Fig. 4). REtarget-generated RNP A1 created a dominant 1 bp insertion at *PPP1R12C* site 1, retargetable by RNP B1 (Supplementary Fig. 4). Efficient depletion of the indel by RNP B1 increased insertion of a 3 bp motif by 2.6-fold in HEK-293T cells and 1.9-fold in K-562 cells (Supplementary Fig. 4). Initiation with RNP A2 produced the same 1 bp insertion and yielded a similar trend when paired with RNP B1 (Supplementary Fig. 4). A second site in *PPP1R12C* (site 2) resulted in two predominant indels and yielded lower HDR improvement (Supplementary Fig. 4). We then aimed to insert large cargo at AAVS1. Using site 1 RNPs A1 and B1, we increased incorporation of a 3.8 kb puromycin-GFP cassette (Fig. 3c). Finally, we attempted to use Recursive Editing to simultaneously knockout TCRα and insert a chimeric antigen receptor (CAR)^29^. A two-level Recursive Editing experiment targeting *TRAC* led to increased incorporation of BCMA-CAR^30^ in CD4 + T cells, accompanied by efficient retargeting of the RNP A1 outcome (Fig. 3d). The overall insertion efficacy was on par with a previously identified gRNA (g526)^30^. Together, our data indicate that Recursive Editing increases the efficiency of inserting larger payloads at select loci.

Next, we used REtarget to query the potential therapeutic utility of Recursive Editing to correct disease-causing genetic mutations. We employed REtarget to find the best Recursive Editing gRNA for each annotated pathogenic mutation in ClinVar, excluding indels >50 bp. We found that 84.3 % (79,363 of 94,142) of human pathogenic mutations fit stringent search criteria for efficacy and specificity (Supplementary Data 3, Supplementary Note 1). To facilitate multiple tests of the resource, we used Recursive Editing to install pathogenic mutations into a wildtype background. A 2 bp frameshift deletion in *ABCA3* (ClinVar ID: 1317554) is linked to neonatal respiratory failure^31^. Using Recursive Editing, we increased HDR-mediated editing from 36.1 ± 0.5 % to 59.1 ± 2.3 % through retargeting of a predominant 1 bp insertion (Fig. 3e). Recursive Editing also increased the editing of a transversion SNP in *ZEB1* (ClinVar ID: 817540) by 40 % relative to RNP A1 (Fig. 3f). This ClinVar-linked Recursive Editing database can serve as an excellent starting point for preclinical work to install and/or revert genetic variants.

### Assessing potential adverse effects of Recursive Editing

**Fig. 4:**
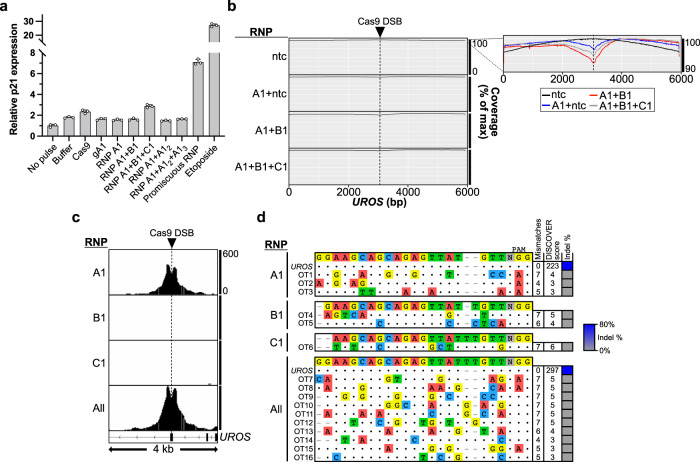
Recursive Editing does not increase deleterious genome editing effects. **a** RPE1 cells (p53 proficient) were electroporated with the indicated reagents and analyzed 24 h later by RT-qPCR for p21 activation. RNP A1, B1, and C1 target *UROS*. RNP A1_2_ and A1_3_ target *PPP1R12C* and *PLCB4*, respectively. The promiscuous RNP targets 14 endogenous target sites^18^. Each data point represents a biological replicate (*n* = 3). Error bars + /− standard deviation (SD). **b** Coverage of PacBio reads containing a deletion at *UROS* with the indicated combinations of RNPs in HEK-293T cells (*n* = 1). The inset panel constitutes an overlay with an adjusted y-axis. The dotted line represents the expected DSB site of *UROS* gA1. **c** On-target DISCOVER-Seq reads at *UROS* with the indicated combinations of RNPs in K-562 cells (*n* = 1). The y-axis denotes the read count. The dotted line represents the expected DSB site of *UROS* gA1. **d** Summarized off-targets (OT) in each sample detected by relaxed parameter DISCOVER-Seq for each sample. Dots correspond to positional matches and dashes account for the change in alignment due to 1 and 2 bp insertions. Source data are provided as a Source Data file.

The increased number of unique gRNAs and Cas9-induced DSBs required for Recursive Editing could theoretically exacerbate deleterious effects of genome editing. We found Recursive Editing with up to three RNPs in p53 proficient RPE1 cells did not lead to transcriptional upregulation of p21, a proxy for p53 activity^32^ (Fig. 4a). We also assessed the formation of large deletions using PacBio long read sequencing at three Recursively Edited loci. We did not observe a substantial increase in large deletions upon the addition of subsequent-level RNPs in the presence of an HDR donor (Fig. 4b, Supplementary Fig. 5, Supplementary Table 1), results which mirror published data^33^. Finally, we performed unbiased off-target identification for Recursive Editing at *UROS* with gRNAs A1, B1, and C1 using DISCOVER-Seq^34^. On-target editing at *UROS* was observed with RNP A1 alone or in the condition containing all three RNPs (Fig. 4c). Potential off-target sites with very high mismatches were only found when relaxing the DISCOVER-seq search parameters (Fig. 4d, Supplementary Table 2). No detectable editing was observed at any off-target using amplicon sequencing. In summary, the addition of one to two additional gRNAs with Recursive Editing does not amplify any negative consequences typically seen with Cas9-mediated genome editing at the tested loci.

## Discussion

Here we introduce Recursive Editing and the companion REtarget software as a strategy to enhance HDR. Harnessing REtarget in silico searches, undesired editing outcomes are systematically retargeted in the presence of a donor DNA template, thereby increasing HDR rates while simultaneously decreasing indel outcomes. Recursive Editing is effective across diverse cell types and applications due to the deterministic nature of indel frequencies across cellular contexts^18^. The flexibility of Recursive Editing HDR means that it encompasses alleles inaccessible to base editing, such as transversions and deletions. While most pathogenic alleles are accessible for prime editing, the generally lower efficiency and large size of the prime editing construct suggests HDR could be a preferred approach. Overall, we expect Recursive Editing to be a broadly applicable technique to enhance HDR.

While the revised version of this manuscript was under review, a method comparable to Recursive Editing, termed double tap, was published^35,36^. The concept of retargeting indels is similar between both methods. However, double tap is an empiric approach requiring prior knowledge of gRNA indel profiles or testing of numerous gRNAs to obtain permissive candidates for retargeting. Lack of this prior knowledge adds a substantial burden to implementing double tap. Recursive Editing minimizes upfront experimental optimization by computationally identifying Recursive Editing sites and generating concrete proposals for cascades of recursive gRNAs. We believe REtarget is a crucial tool that facilitates the efficaciousness of Recursive Editing to simultaneously increase HDR and decrease error-prone indels.

Successful implementation of Recursive Editing depends upon the accurate prediction of editing outcomes and gRNA efficacy. We found the magnitude of retargeting of the primary indel correlates with the degree of HDR improvement (r = 0.77, Pearson correlation coefficient; Supplementary Fig. 6a, Supplementary Table 3). Importantly, Recursive sites, defined as having >20% retargeting efficiency, exhibit substantial increases in HDR, whereas non-Recursive sites (<20% retargeting efficiency) mostly do not benefit from additional gRNAs (Supplementary Fig. 6i). This relationship was not influenced by the type of initial indel produced, whether an NHEJ or alt-EJ (≥3 bp deletion) outcome. We did not, however, observe a positive correlation between the REtarget score and HDR enhancement (r = −0.39). Consequently, the REtarget score cannot be used to quantitatively predict the extent of HDR improvement. We instead recommend using REtarget scores as priority metrics for locus-specific Recursive Editing reagents.

A disconnect between predicted and experimental gRNA efficacy suggests that improved on-target metrics for individual gRNAs could also drive improved Recursive Editing HDR. We compared our empirical efficiency data for multiple gRNAs to three different prediction algorithms and observed little correlation^23,37,38^. Further advances in the accuracy and breadth of gRNA efficacy prediction could be easily incorporated into future versions of REtarget. While REtarget is not intended to set expectations for overall HDR, REtarget and Recursive Editing were able to consistently enhance the HDR frequency and HDR:indel ratio (Supplementary Fig. 6). We anticipate the pre-calculated Recursive Editing databases focusing on overall efficiency, protein tagging, and pathogenic mutations will be useful tools for genome editors with diverse experimental goals.

Looking forward, Recursive Editing could potentially be combined with more aggressive HDR enhancement strategies such as donor modifications^39,40^ or Cas9 fusions^12,13,15^ to further boost precise editing outcomes. The target space amenable for Recursive Editing can be expanded by harnessing PAMless Cas9 variants such as SpRYCas9^41^ or incorporating potential indel prediction algorithms for other nucleases such as Cas12a. REtarget also possesses the capability to search any user-defined genomic sequence. Overall, Recursive Editing could be a useful and effective tool for increasing the efficacy of gene modification in both research and clinical settings.

## Methods

### Nucleotide sequences and cloning

All nucleotide sequences – including gRNA, donor, and oligonucleotide sequences – can be found in Supplementary Tables 4–6. ssODNs were designed with 40–50 bp homology arms containing a 3-bp insertion (5′-GAT-3′) at the cut site of the gRNA A1 with three phosphorothioate modifications each at the 5′ and 3′ ends, except where noted. DNA was purchased from Integrated DNA Technologies (IDT) and Microsynth AG. Synthetic gRNAs were purchased from Synthego and IDT. Plasmids were purchased from Addgene.

### Cell culture

Cell lines were obtained from ATCC or Berkeley Cell Culture and were STR profiled. K-562 cells were cultured in RPMI medium (Gibco), 10% fetal bovine serum (FBS), and 100 µg / mL penicillin/streptomycin. HEK-293T, HCT-116, and RPE1 cells were cultured in Dulbecco’s modified Eagle’s medium (DMEM, Gibco), supplemented with 10% FBS and 100 µg / mL penicillin/streptomycin. All cells were incubated at 37 °C and 5% CO_2_. Cell lines were routinely tested for mycoplasma contamination (MycoAlert; Lonza) and tested negative.

### T cell isolation and culturing

CD4 + T cells were purified from frozen human peripheral blood Leukopak (StemCell) by negative selection using the EasySep Human T Cell Enrichment Kit (StemCell) according to the manufacturer’s instructions and cryopreserved in CryoStor CS5 (StemCell). Purified T cells were cultured in X-VIVO 15 Media (Lonza) supplemented with 5% human AB serum (GeminiBio) and 100 IU / mL human IL-2 (Miltenyi Biotec). For *UROS* editing, T cells were activated using TransAct (Miltenyi Biotec) according to the manufacturer’s instructions. The activated cells were used in electroporation experiments 6 days post-activation as described below. For *TRAC* editing, T cells were activated one-day post-thaw using CD3/CD28 Dynabeads (Thermo Fisher) per the manufacturer’s protocol. Two days after bead addition, the beads were removed using magnetic separation. The T cells were used in experiments either day three or four post-thaw.

### In vitro transcription

gRNAs were produced in vitro using previously published methods^42^. Briefly, overlapping oligomers containing a T7 promoter, the desired protospacer, and gRNA scaffold were amplified using Phusion polymerase (New England Biolabs). The unpurified DNA product was then subjected to in vitro transcription using the NEB HiScribe T7 High Yield RNA Synthesis Kit (New England Biolabs), incubating at 37 °C for 16 h. The following day, RNA was treated first with DNase I followed by rSAP (recombinant Shrimp Alkaline Phosphatase; New England Biolabs), purified with the miRNeasy kit (Qiagen), concentration measured by Nanodrop, and frozen at −80 °C.

### Production of double-strand DNA donors

Double-strand DNA donors were amplified from plasmid DNA using Q5 High-Fidelity DNA Polymerase (New England Biolabs) per the manufacturer’s protocol. Common PCR amplicons were pooled, SPRI purified (1x), and eluted in water. The concentration was measured by Nanodrop, and the length was confirmed by agarose gel electrophoresis. The plasmid template of each dsDNA donor are as follows: HIST1H2BJ-mEGFP (Addgene #109121; a gift from the Allen Institute for Cell Science), FBL-mEGFP (Addgene #87427; a gift from the Allen Institute for Cell Science), RAB11A-GFP (Addgene #112012; a gift from Alexander Marson), AAVS1-CAG-hrGFP (Addgene #52344; a gift from Su-Chun Zhang), and BCMA-CAR (a gift from Alexander Marson). A SNP to prevent *TRAC* RNP A1 from cutting the BCMA-CAR donor was incorporated using site directed mutagenesis. The modified donor sequence is available in Supplementary Table 4.

### RNP electroporation

All experiments were conducted with in-house produced SpCas9-NLS (40 µM) using previously published protocols^43^. For RNP formation, SpCas9-NLS, gRNA, and Cas9 buffer (20 mM HEPES, pH 7.5, 150 mM KCl, 1 mM MgCl_2_, 10 % glycerol, and 1 mM TCEP) were mixed with a ratio of 1:1.2 (Cas9:gRNA) and incubated for 5–10 min. 50 pmol of each RNP was used. For experiments with 2 RNPs, this equals 100 pmol total. For experiments with ≥3 RNPs, the total RNP amount was therefore above 100 pmol. Regardless of the amount of RNP, 100 pmol of ssODN was then added. For reactions using double-strand donors, 700 ng of DNA was added. For reactions using plasmid donors, 1500 ng of DNA was added. Transfections were conducted in 96-well format (2 × 10^5^ cells per well) or with 100 µL cuvettes (1 × 10^6^ cells per cuvette) using Lonza 4D electroporation kits. The kit / program for each cell type was as follows: K-562 (SF kit / FF-120), HCT-116 (SE kit / EN-113), HEK-293T (SF kit / DG-130), T cells (P3 kit / EH-115), and RPE1 (P3 kit / EA-104). After electroporation, cells were allowed to sit at RT for 10 min before incubating with 2 mL medium per 2 × 10^5^ cells in a 6 well-plate for all cell types except T cells. For T cells, 2 × 10^5^ cells were cultured in 96 well-plate format. For *TRAC* editing in T cells, 0.8 µL of 100 mg / mL of 15,000–50,000 kDa poly-L-glutamic acid (Sigma) was supplemented after gRNA addition. For single-electroporation experiments, cell pellets were collected after 72 h. For sequential experiments, the second transfection was carried out 48 h after the initial electroporation using 2 × 10^5^ cells of the recovered population. Cell pellets were then collected a further 72 h later.

### Genomic DNA extraction

Cell pellets (collected above) were washed with PBS and resuspended in QuickExtract solution (Lucigen). Reactions were incubated as follows: 10 min at 65 °C, 5 min at 98 °C, hold at 4 °C. Following incubation, samples were spun down, and the clarified supernatant was used for downstream analysis. For PacBio long-read sequencing, genomic DNA was purified using the DNeasy Blood and Tissue kit (Qiagen).

### Sanger sequencing

Primers were designed to yield 500–700 bp amplicons surrounding the predicted cut site. Genomic DNA was amplified by Q5 (Q5 High-Fidelity DNA Polymerase, New England Biolabs) with Q5 GC enhancer. Amplicons were analyzed by agarose gel electrophoresis. Samples containing a single clean band were subsequently purified without running on a gel using QIAquick PCR purification kit (Qiagen). Samples requiring gel extraction were gel purified (Zymoclean Gel DNA Recovery Kit; Zymo Research). Purified samples were Sanger sequenced (Microsynth AG) and the resulting traces were deconvoluted and analyzed using ICE^44^.

### Illumina amplicon sequencing

Primers were designed to amplify a 150–250 bp region surrounding the cut site. Genomic DNA was amplified in two rounds using NEBNext Ultra II Q5 HiFi polymerase (New England Biolabs). In the first round, Illumina adapter sequences were included at the ends of the primers amplifying the genomic DNA in 20 reaction cycles. In the second round, 1 µL from the first PCR was used as input and i7 / i5 Illumina indexes were added in 10 reaction cycles. Common amplicons were then pooled and purified using 0.8x SPRI beads (SeraMag; Cytiva). Concentrations were measured on a Qubit and further analyzed for amplicon length and sample purity on a TapeStation high-sensitivity DNA flow cell (Agilent). Pools were then normalized and combined. The combined samples were sequenced either with a MiSeq 2 × 100 paired-end or a NextSeq2000 2 × 150 paired-end (Illumina) by the Functional Genomics Centre Zurich (FGCZ) in combination with the Genome Engineering and Measurement Lab (GEML) at ETH Zurich. The target average read count per amplicon was 100–200k reads.

### Analysis of Illumina sequencing

Illumina reads were demultiplexed and analyzed with Crispresso2 (v2.0.20b) in batch mode^45^. The non-default parameters were minimum average read quality of 30 and minimum single bp quality of 20. For the remaining parameters, default settings were used. Reads with a frequency lower than 0.5% were disregarded before further analysis. Results were then normalized to sum up to 100%.

### Flow cytometry

Five to seven days postelectroporation, K-562 cells were pelleted, washed once with PBS, resuspended in a PBS / 2% FBS solution, and analyzed immediately. For cell surface antibody staining, T cells were pelleted 3 days post-electroporation, resuspended in PBS / 2% FBS solution containing antibody, and incubated 10–30 min at RT. Cells were then pelleted, washed once, and resuspended for analysis. The following antibodies were used: 1:100 dilution of Brilliant Violet 421 anti-human TCRα/β (Clone IP26; BioLegend 306722) and 1:50 of Alexa Fluor 647 anti-myc tag (Clone 9B11; Cell Signaling 2233). Samples were analyzed using an Attune NxT Flow Cytometer (Thermo Fisher), software v3.2.1. Data were analyed in FlowJo (v10.8) The gating strategy is exemplified in Supplementary Fig. 7. Live, single cells were gated on SSC-A versus FSC-A then FSC-A versus FSC-H. GFP HDR was assessed by gating on SSC-A versus GFP. CAR HDR was assessed by gating on Brilliant Violet 421 (TCRα/β) versus Alexa Fluor 647 (myc-CAR).

### Reverse transcription quantitative PCR (RT-qPCR)

RPE1 cells were electroporated as described above or incubated with 100 µM etoposide (Sigma-Aldrich). 24 h later, cellular RNA was isolated using the RNeasy kit (Qiagen) with on-column DNase digestion. RNA concentration was measured by Nanodrop, and 125 ng was used as input for the subsequent reverse transcription (iScript cDNA Synthesis Kit; Bio-Rad). qPCR was then performed with a 1:10 dilution of cDNA using SsoAdvanced Universal SYBR Green Supermix (Bio-Rad) on a QuantStudio 6 (Thermo Fisher) per manufacturer’s protocol. Data was then analyzed by the ddCt method using β-actin as the endogenous control.

### PacBio long read sequencing

Primers were designed to amplify a ~6 kb region surrounding the expected cut site. 600 ng of genomic DNA (corresponding to ~1 × 10^5^ cells) was amplified using Q5 (Q5 High-Fidelity DNA Polymerase, New England Biolabs) with an annealing temperature of 60 °C, extension time of 3 min 30 s, and a cycle number of 30. Common amplicons were then pooled and purified using 0.5x SPRI beads (Sera-Mag; Cytiva). Purified amplicons were then given to Functional Genomics Centre Zurich (FGCZ) for indexing. Indexed amplicons were analyzed on a PacBio Sequel IIe with a SMRT Cell 8 M, 30 h movie.

### Analysis of PacBio sequencing

FGCZ demultiplexed reads and collapsed subreads to high-quality Circular Consensus Sequence (CCS) reads. CCS reads were mapped to their respective amplicon reference sequence with the Mapping application in SMRT Link (v10.2.0.133434) with default parameters: Minimum CCS Predicted Accuracy (Phred Scale): 20; Minimum Gap-Compressed Identity (%): 70; Minimum Mapped Length (bp): 50. Depth of coverage data were extracted from the resulting BAM files using bedtools genomcov (v2.27.1) with the -dz flag and smoothed using the mean coverage in a 10 bp sliding window using custom R code. Coverage plots were generated using ggplot2 in R. Data from long repetitive regions (i.e. polyT stretches) were filtered out where the coverage dropped due to mapping issues. Three base insertions from HDR donors were identified using callvariants2.sh from BBMAP (v38.69).

### DISCOVER-Seq

20 × 10^6^ K-562 cells were mixed with 400 pmol of each RNP and electroporated with 10 × 10^6^ per 100 µL cuvette as described above. The two cuvettes for each condition were allowed to rest following electroporation for 10 min and then combined in a T-75 flask, adding media to bring the cells to a density of 0.8 × 10^6^ cells per mL. 12 h post-electroporation, cells were pelleted and resuspended in room temperature RPMI (without supplements). Cells were crosslinked with 1 % formaldehyde (Thermo Fisher) and incubated for 15 min at room temperature. Formaldehyde was quenched with 125 mM glycine for 3 min on ice. Cells were then pelleted at 4 °C, washed twice with ice-cold PBS, and snap frozen in liquid nitrogen. Pellets were stored at −80 °C prior to processing.

MRE11 ChIP-Seq was next performed^34^. Briefly, samples were thawed on ice and lysed using ice-cold buffers LB1, LB2, and LB3. The isolated DNA was sonicated to obtain ~300 bp chromatin fragments using a Covaris S2 with the following settings: 12 cycles of duty cycle 5 %, intensity 5, 200 cycles per burst for 60 s. 10 μL of MRE11 antibody (NB 100-142; Novus Biologicals) per ChIP was prebound to protein A Dynabeads (Invitrogen). Chromatin were immunoprecipitated with antibody-bound beads, rotating overnight at 4 °C. Dynabeads were washed with RIPA buffer and the DNA was eluted by incubating overnight at 65 °C in elution buffer containing. For the final clean-up, the samples were digested with Proteinase K and RNase A in TE buffer for 1 h at 55 °C. DNA fragments were purified using the MinElute Kit (Qiagen). Sequencing libraries were prepared using NEBNext Ultra II kit (New England BioLabs). Indexed libraries were pooled and sequenced on a NextSeq2000 (Illumina) with 2×150 paired-end reads and a target depth of 20 M reads per sample. Bowtie2 (v2.4.5) was used to align the reads, and off-target peak calling was performed using BLENDER with relaxed parameters^34^. Off-targets with less than seven mismatches, or ones with seven mismatches with a DISCOVER score ≥3, were assigned an off-target number and further validated with targeted Illumina amplicon sequencing. For some targets, nested PCR was used (Supplementary Table 2).

### REtarget database generation

For the genome-wide search for sites amenable to Recursive Editing (Supplementary Data 1), we downloaded sequences of all human chromosomes (GRCh38) from GenBank (accessed 10/2021). For the start and stop codon database (Supplementary Data 2), we extracted every RefSeq entry (accessed 12/2021) annotated as start_codon or stop_codon. Redundant entries that overlapped the same start/stop codon because of different gene or isoform names were filtered out. For the ClinVar comparison (Supplementary Data 3), the ClinVar variant summary was downloaded from NCBI (accessed 11/2021). The summary list was filtered to only contain pathogenic variants. Of the pathogenic variants, ones with an indel >50 bp were filtered out, leaving a list of 94,130 mutations. The genomic coordinates of these mutations were used as input for REtarget, where a ± 50 bp window was searched for potential Recursive Editing sites. Exact parameters applied to run REtarget for each database can be found in Supplementary Note 1.

### Software

In addition to the aforementioned software, we used the following tools in the course of this study. inDelphi^22^ (v0.18.1) and Lindel^20^ (version as of 05/2021) were downloaded from GitHub and ran locally to predict editing outcomes. On-target editing efficiencies were predicted using Doench 2014 scores as implemented in the CRISPOR (v4.99) package^23,46^. Off-target scoring was performed using FlashFry (v1.11)^24^. REtarget was implemented in Python (v3.7.10), tested on a Linux operating system, and requires the modules Biopython (v1.78), Kiwisolver (v1.3.1), Matplotlib (v3.2.2), Numpy (v1.15.3), Pandas (v0.23.4), Scikit-learn (v0.20.0) and Scipy (v1.1.0). The online version of REtarget was built with Dash (v2.0.0) / Plotly (v5.3.1). All graphs were produced in Prism (v9.3.1; GraphPad), and figures were assembled using Affinity Designer (v1.10.5; Serif).

### Statistics and reproducibility

All bar graphs demonstrate the mean ± standard deviation. All biological replicates were performed at independent time points. No statistical methods were used to determine the sample size or required number of replicates. No data were excluded from the analyses except in cases of failed experiments due to other factors (reagent loss of function, contamination, etc). The experiments were not randomized. The Investigators were not blinded to allocation during experiments and outcome assessment due to the non-subjective nature of the results. Pearson correlation coefficients were calculated for Supplementary Fig. 6a,c-f and a two-sided Mann-Whitney U Test was performed for Supplementary Fig. 6i.

### Reporting summary

Further information on research design is available in the Nature Research Reporting Summary linked to this article.

## Supplementary information

Supplementary Information

Reporting Summary

Supplementary Data 1

Supplementary Data 2

Supplementary Data 3

Description of Additional Supplementary Files

## Data Availability

GRCh38 was downloaded from GenBank. Sequencing data is deposited in SRA BioProject PRJNA837763. The REtarget-generated databases are available as Supplementary Data 1–3. Source data are provided with this paper.

## Source data

Source Data

## Code availability

The web-based version of REtarget is free for use by nonprofit users and can be accessed at https://recursive-editing.herokuapp.com/ (Supplementary Fig. 8). The underlying REtarget software is also immediately available for free to nonprofit users by contacting Dr. Jacob Corn (jacob.corn@biol.ethz.ch). Requests will be responded to within 1 week, barring unforeseen circumstances. REtarget is available for license to for-profit groups by contacting either Dr. Jacob Corn or ETH Transfer (emanuel.weber@sl.ethz.ch). We recommend running the website in Chrome or Firefox. Note the online version of REtarget does not incorporate off-target search; rather, the user can follow the provided links to CRISPOR^46^ or CasOFFinder^47^. Further detailed information on REtarget can be found in Supplementary Note 1.
