# Supplementary material for: Recursive Editing improves homology-directed repair through retargeting of undesired outcomes
Source: Nat Commun. 2022 Aug 5;13:4550. doi: 10.1038/s41467-022-31944-7 (PMC9356142; doi:10.1038/s41467-022-31944-7)
Supplement: Supplementary file 1 — Supplementary Information [file 41467_2022_31944_MOESM1_ESM.pdf]

Supplementary Information for:

Recursive Editing improves homology-directed repair through retargeting of undesired outcomes

Lukas Möller[1], Eric J. Aird[1]*, Markus S. Schröder[1], Lena Kobel[1], Lucas Kissling[1,2], Lilly van de Venn[1], Jacob E. Corn[1]*

[1] Institute of Molecular Health Sciences, Department of Biology, ETH Zurich. Zurich, Switzerland.
[2] Institute of Pharmacology and Toxicology, University of Zurich. Zurich, Switzerland.

*Correspondence: eric.aird@biol.ethz.ch (EJA), jacob.corn@biol.ethz.ch (JEC)

Table of contents:

Supplementary Figure 1. Comparison of top-ranking predictions and experimental data.
Supplementary Figure 2. Extended editing data at *UROS*.
Supplementary Figure 3. Inefficient retargeting reduces the degree of HDR improvement.
Supplementary Figure 4. Efficient ssODN-mediated Recursive Editing at *PPP1R12C.*
Supplementary Figure 5. Assessing large deletions at more Recursive Editing sites.
Supplementary Figure 6. Recursive Editing summary.
Supplementary Figure 7. Flow cytometry gating scheme.
Supplementary Figure 8. User interface of the online version of REtarget.
Supplementary Table 1. PacBio deletion data.
Supplementary Table 2. DISCOVER-Seq off-target information.
Supplementary Table 3. Aggregated HDR data.
Supplementary Table 4. gRNA sequences.
Supplementary Table 5. HDR donor sequences.
Supplementary Table 6. PCR primer sequences.
Supplementary Note 1
- Extended discussion of REtarget scores
- Theory underlying Recursive Editing
- Algorithmic scheme of REtarget to predicts suitable gRNAs for Recursive Editing
- gRNA filtering criteria of REtarget
- REtarget syntax to describe Recursive Editing and associated gRNAs
- Usage of REtarget web tool
- Explanation of REtarget search parameters
- Explanation of REtarget result files
- Genome-wide search for loci amenable to Recursive Editing
- Search of ClinVar database for loci amenable to Recursive Editing
- Genome-wide start and stop codon search for loci amenable to Recursive Editing

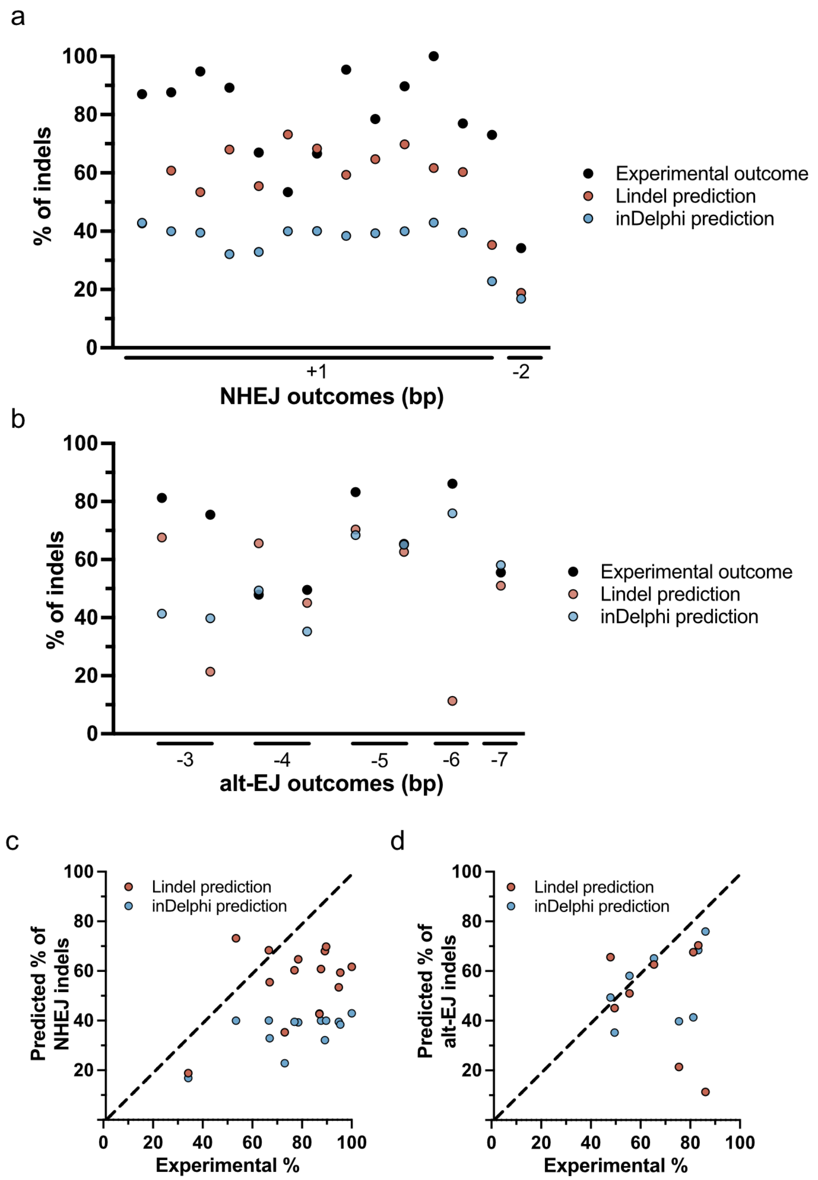

Supplementary Figure 1 | Comparison of top-ranking predictions and experimental data. Comparing predicted editing outcome percentages to experimentally determined outcomes in HEK-293T cells for **a**) loci with predominant NHEJ outcomes (1 bp insertion to 2 bp deletion) and **b**) loci with predominant alt-EJ outcomes (≥3 bp deletion). **c**) Data in **a** and **b** are graphed as the comparison of predicted % of outcome versus the experimental outcome for each target site. The dotted line corresponds to x=y. Source data are provided as a Source Data file.

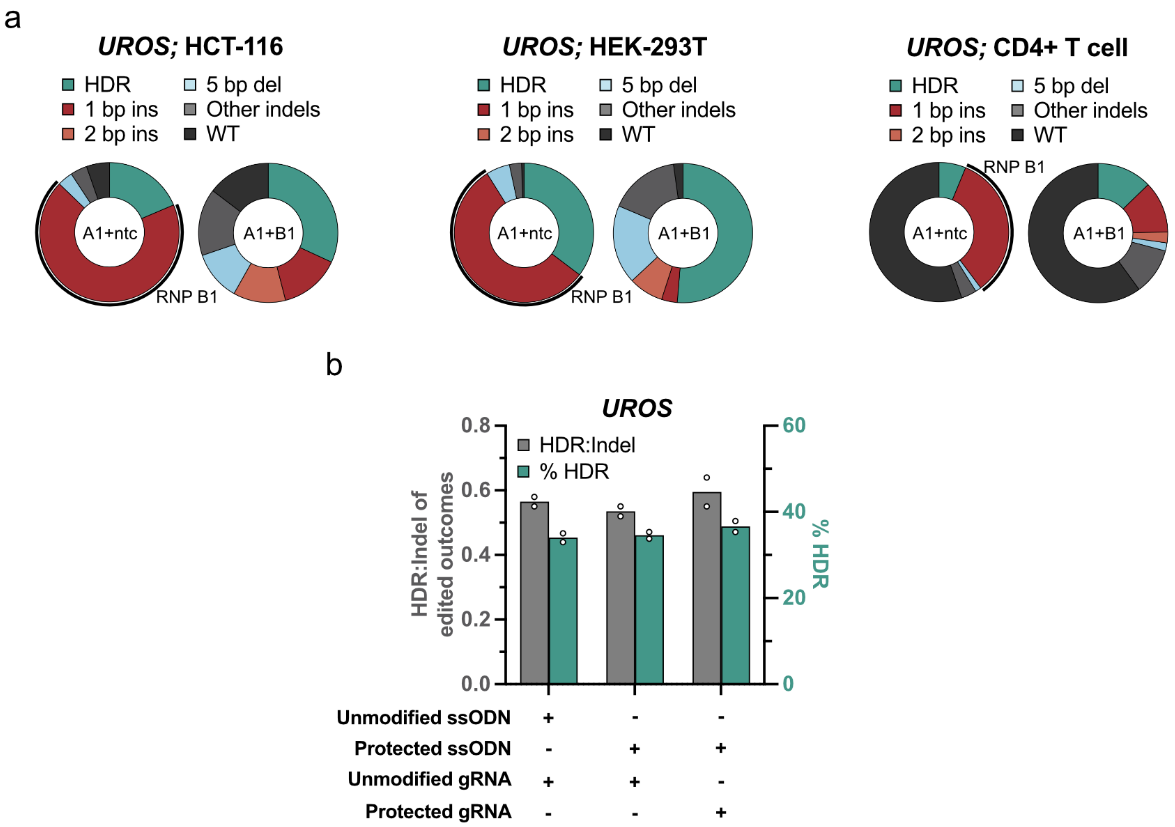

Supplementary Figure 2 | Extended editing data at *UROS*. a) Individual editing outcomes of *UROS*-targeting Recursive Editing RNPs in the indicated cell types. These data reference back to **Fig. 1f**. **b**) Simultaneous editing in *UROS* with gRNAs A1 and B1, either *in vitro* transcribed (unmodified) or chemically synthesized (protected) in the presence of ssODN (either 5’ and 3’ 3x phosphorothioate bonds protected or unmodified) in K-562 cells. Each data point represents an individual biological replicate (n=2). Error bars +/- standard deviation (SD). Abbreviations: ins=insertion, del=deletion; ntc=non-targeting control; indels = insertions and deletions; WT=wildtype. Source data are provided as a Source Data file.

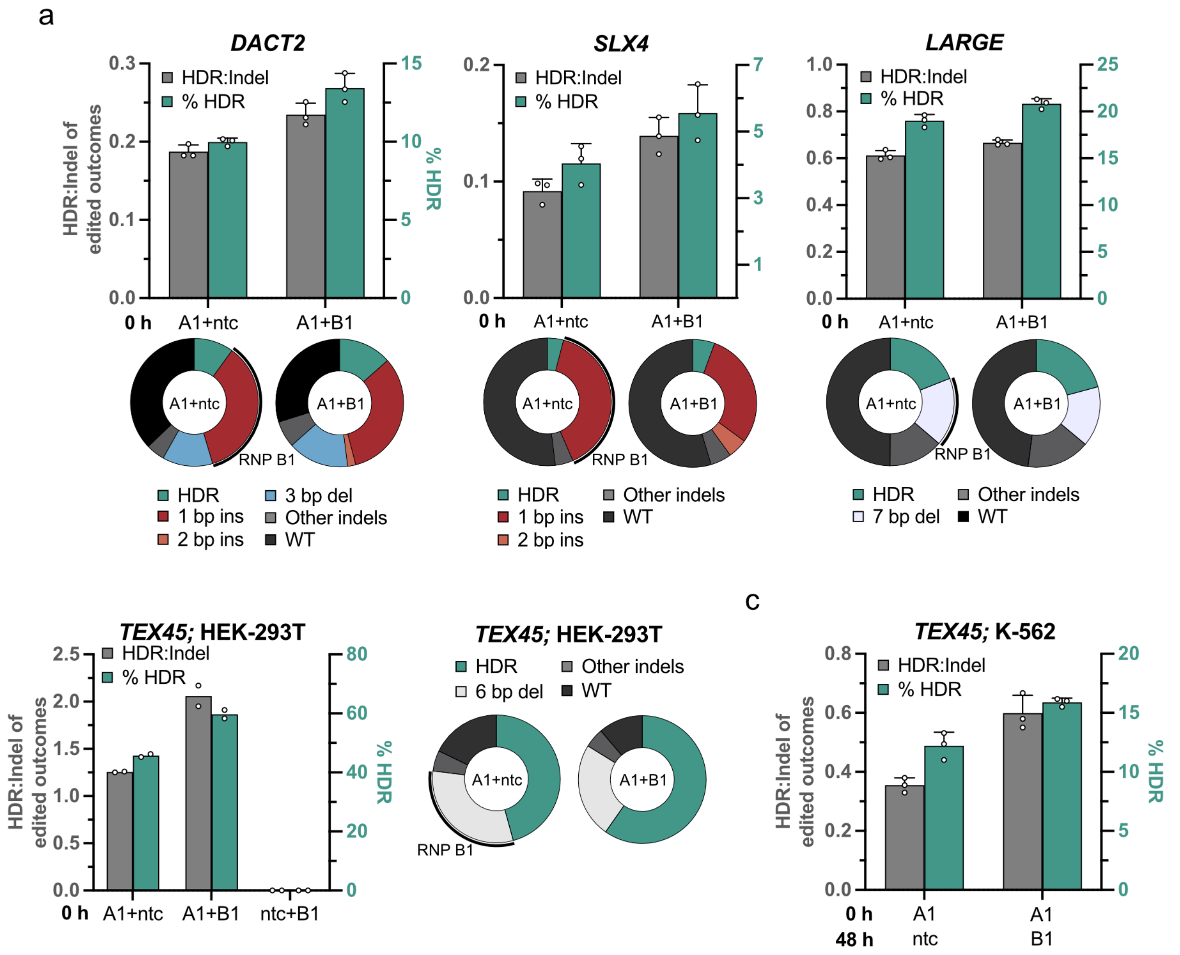

Supplementary Figure 3 | Inefficient retargeting reduces the degree of HDR improvement. **a**) Top panels: Recursive Editing at the indicated target sites in HEK-293T cells. The left y-axis is displayed as HDR:indel and the right y-axis in % HDR. Bottom panels**:** Individual editing outcomes. **b**) Recursive Editing at *TEX45*. Editing in HEK-293T cells was simultaneous (left panel). The associated individual editing outcomes are displayed in the right panel. **c**) Sequential delivery of Recursive Editing reagents in K-562 cells. Each data point represents an individual biological replicate (n=2-3). Axes are scaled for each cell graph. Error bars +/- standard deviation (SD). Abbreviations: ins=insertion; del=deletion; ntc=non-targeting control; indels = insertions and deletions; WT=wildtype. Source data are provided as a Source Data file.

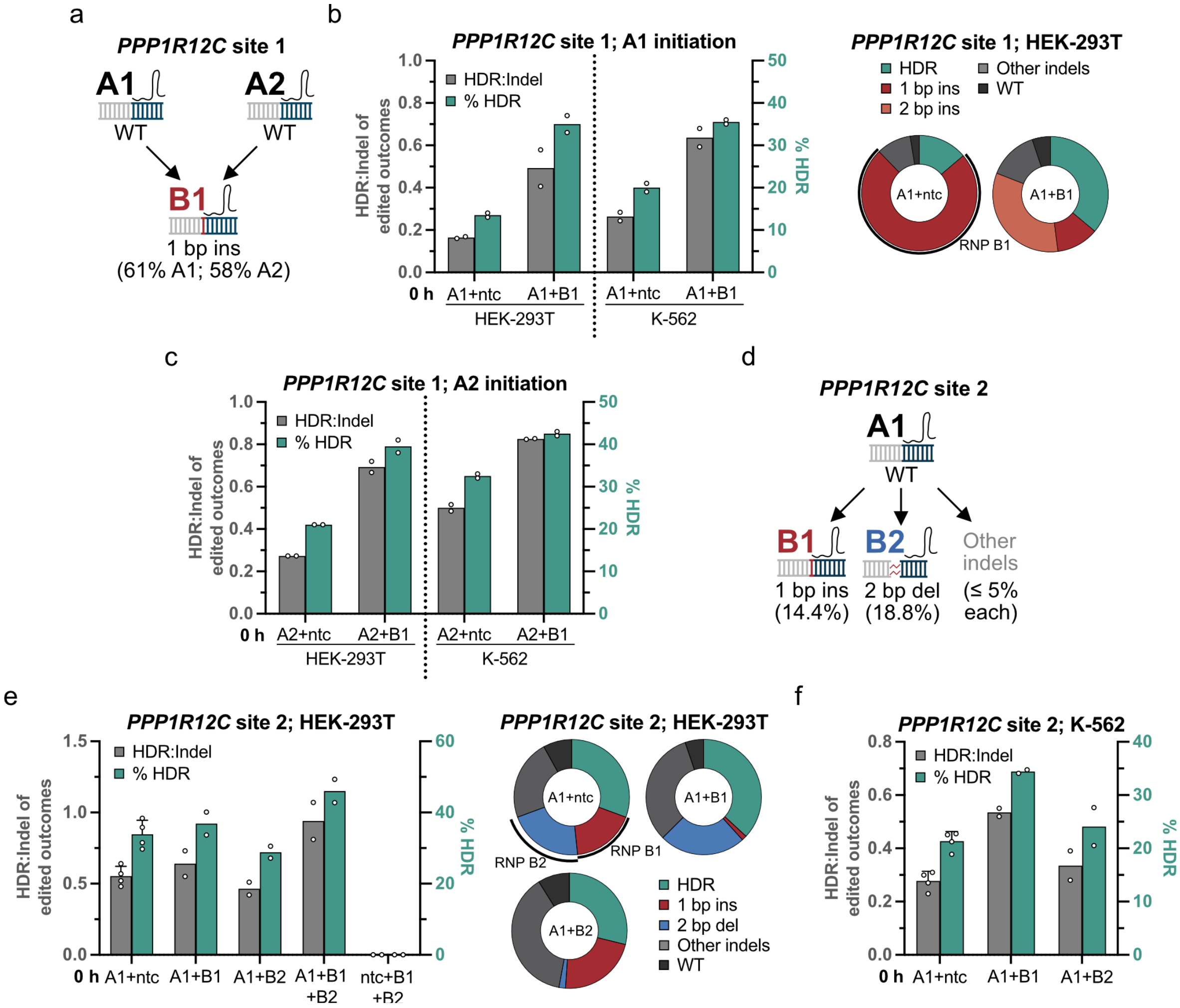

Supplementary Figure 4 | Efficient ssODN-mediated Recursive Editing at *PPP1R12C*. a) REtarget-produced editing tree for *PPP1R12C* site 1. Editing with either gRNA A1 or A2 generates the identical 1 bp ins with the given Lindel predictions. **b**) Simultaneous delivery of Recursive Editing RNPs in the indicated cell types in *PPP1R12C* site 1, initiating with RNP B1. The HDR:indel ratio is displayed on the left y-axis and the corresponding HDR frequency on the right y-axis. Individual outcomes from simultaneous targeting of *PPP1R12C* site 1 in HEK-293T cells with the given gRNA combinations is shown in the right panel. **c**) Simultaneous delivery of Recursive Editing RNPs in the indicated cell types in *PPP1R12C* site 1, initiating with RNP B2. **d**) REtarget-produced editing tree for *PPP1R12C* site 2. Two predominant Lindel-predicted outcomes are made. **e**) Left panel: HDR:indel and % HDR from simultaneous editing at *PPP1R12C* site 2 in HEK-293T cells. Right panel: Individual editing outcomes. **f**) Recursive Editing at *PPP1R12C* site 2 in K-562 cells. Each data point represents an individual biological replicate (n=2-4). Error bars +/- standard deviation (SD). Abbreviations: ins=insertion; del=deletion; ntc=non-targeting control; WT=wildtype. Source data are provided as a Source Data file.

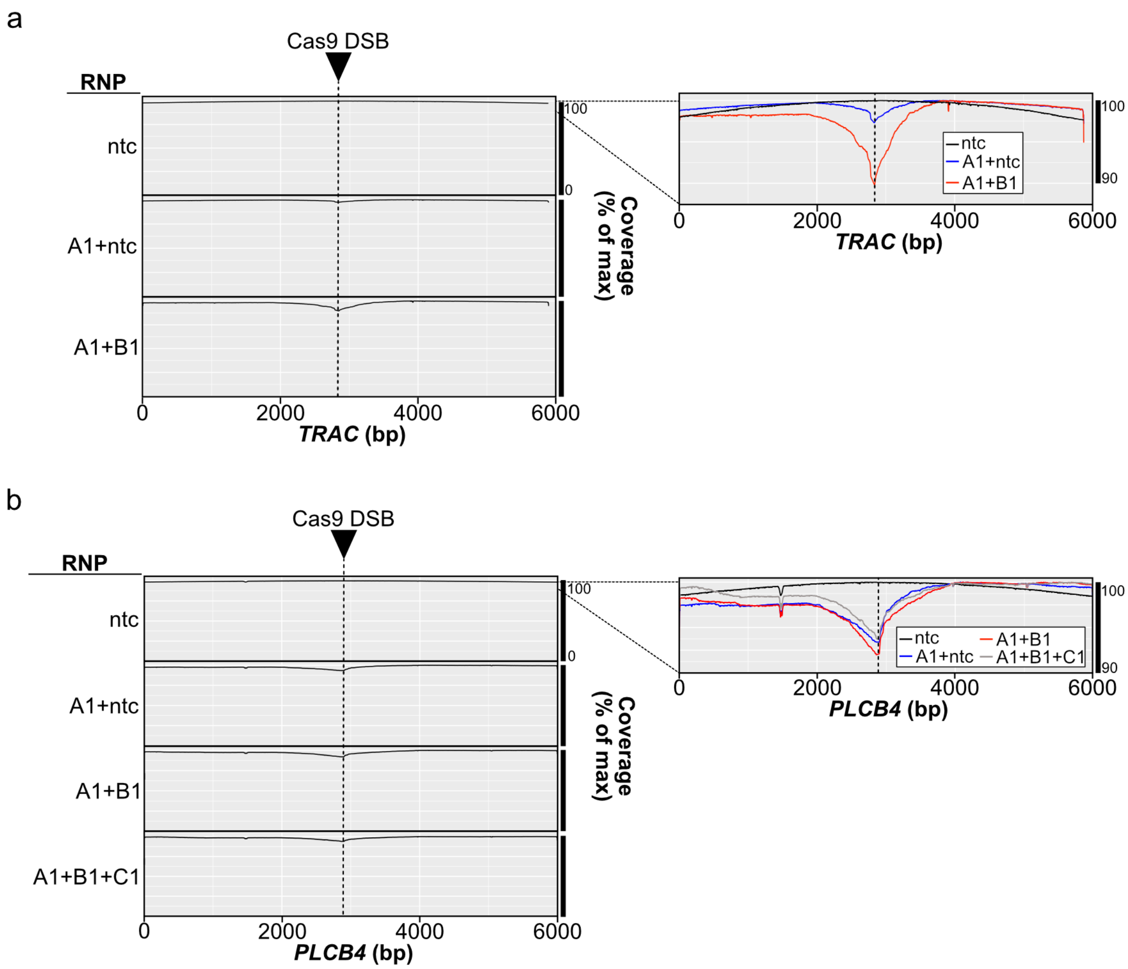

Supplementary Figure 5 | Assessing large deletions at more Recursive Editing sites. Coverage of PacBio reads containing a deletion at **a**) *TRAC* and **b**) *PLCB4* with the indicated combinations of RNPs (n=1). The inset panel constitutes an overlay with an adjusted y-axis. The dotted line represents the expected DSB site of RNP A1.

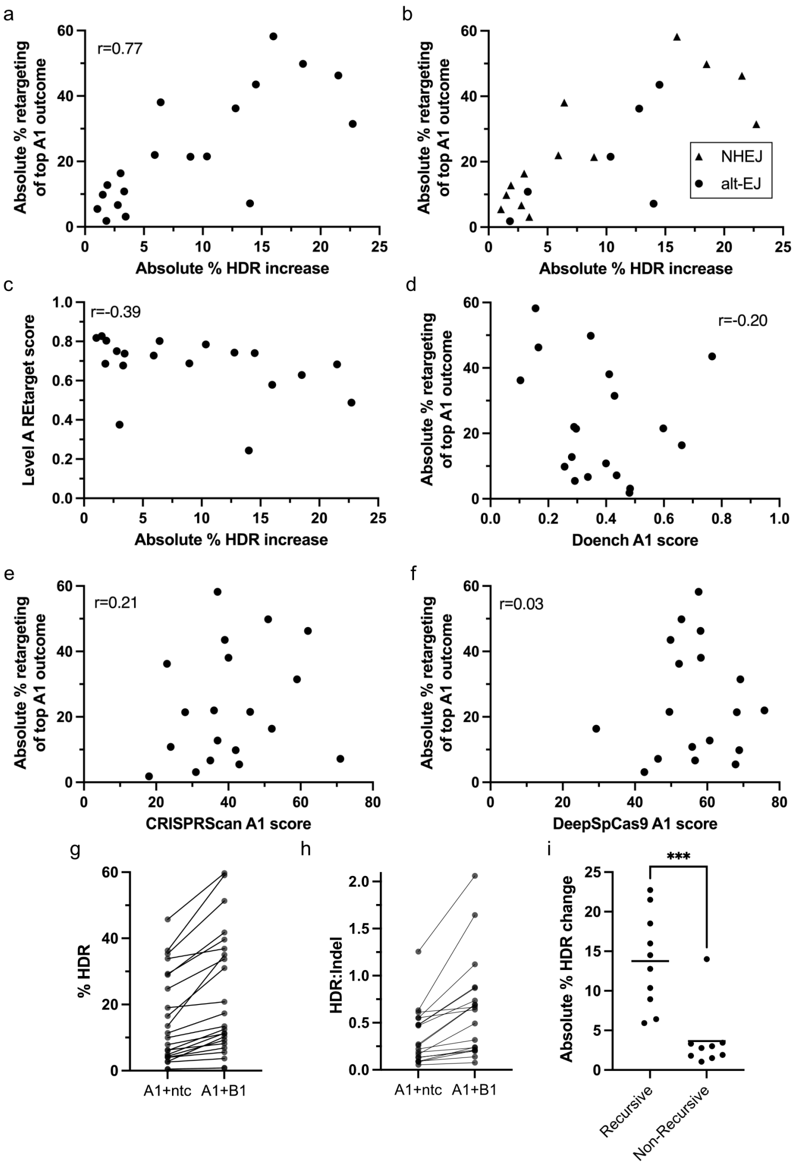

Supplementary Figure 6 | Recursive Editing summary. a) The absolute % retargeting of the top A1 outcome versus absolute % HDR increase in HEK-293T cells (n=18)**.** The absolute % corresponds to the change in abundance from RNPs A1+ntc to RNPs A1+B1. The r-value denotes the Pearson correlation coefficient. **b**) Same data as in **a**, but with points labeled by the type of indel produced, either an NHEJ outcome (1 bp insertion to 2 bp deletion; triangle) or an alt-EJ outcome (≥ 3 bp deletion; circle). **c**) Same x-axis as in **a**, but graphed against the editing level A REtarget score. **d-f**) Comparing the absolute % retargeting of the top A1 outcome to three different gRNA efficacy prediction metrics: **d**) Doench 2014 score, **e**) CRISPRScan score, and **f**) DeepSpCas9 score. **g**) Matched change in % HDR from RNPs A1+ntc to A1+B1 for each locus tested in this manuscript (n=22). For loci tested in multiple cell types, only HEK-293T data are shown. For GFP insertion, data from K-562 cells are shown. **h**) Matched change in HDR:Indel from RNPs A1+ntc to A1+B1 for each locus tested with an ssODN donor in this manuscript (n=18). **i**) Comparison of the average HDR from sites that are Recursive (*i.e.* have > 20% retargeting of the primary indel) versus non-Recursive sites with low retargetability (< 20%) (n=18). The mean is denoted by the horizontal bar. ***: p = 0.0004, two-sided Mann-Whitney U Test. Source data are provided as a Source Data file.

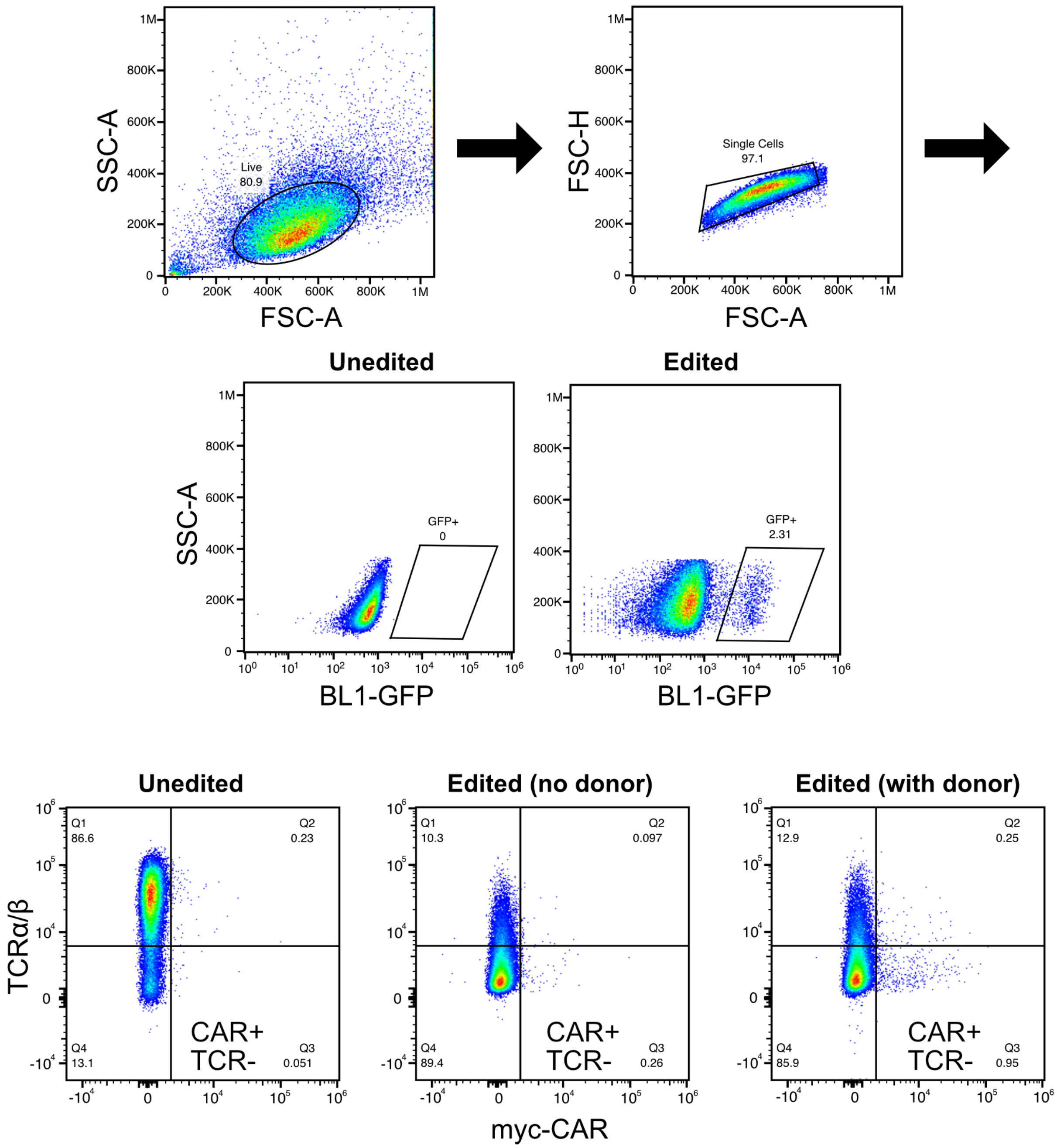

Supplementary Figure 7 | Flow cytometry gating scheme. Top panel: Live cells are first gated on SSC-A versus FSC-A, then single cells are gated on FSC-H versus FSC-A. **Middle panel**: GFP positive cells are then gated on SSC-A versus BL1-GFP. This strategy was used for Figure 3a,c. **Bottom panel**: Starting from the top panel, chimeric antigen receptor (CAR) positive cells and T cell receptor (TCR) negative are gated on TCRα/β-Brilliant Violet 421 vs myc-CAR-Alexa 647 (lower right quadrant; Q3). This strategy was used for Figure 3d.

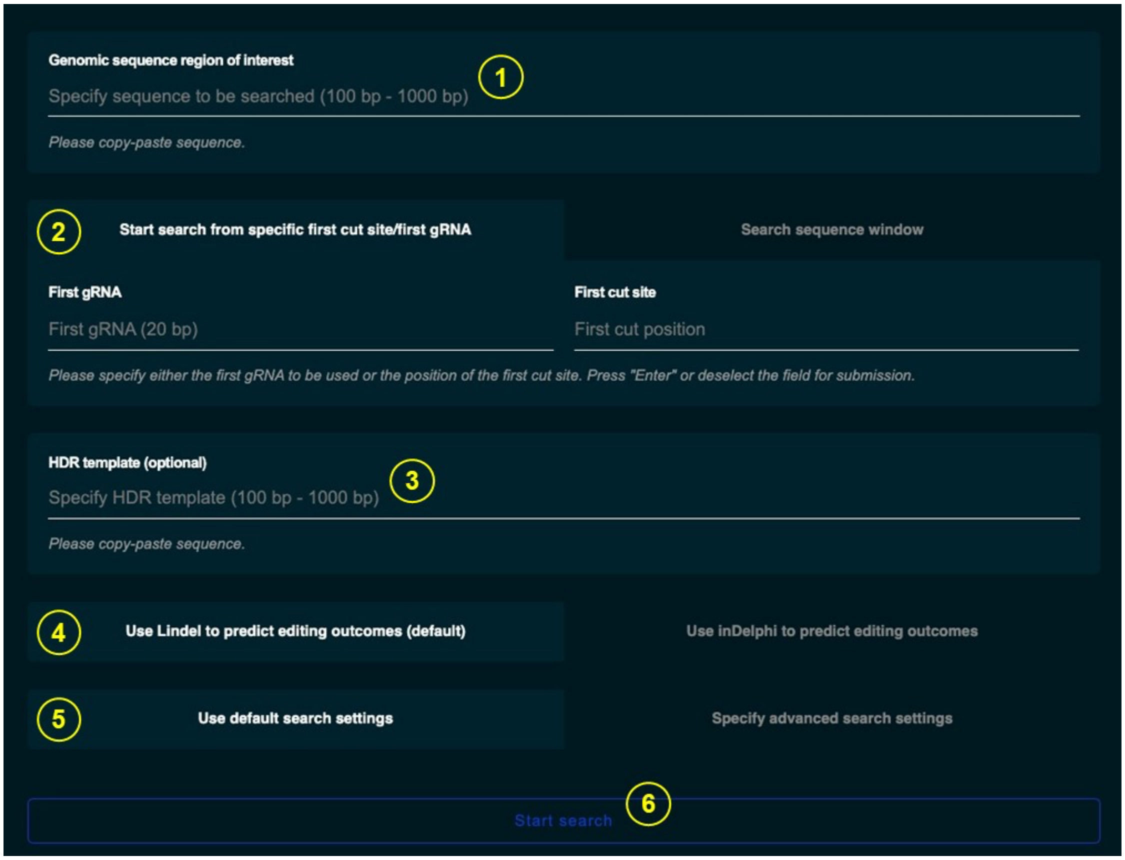

Supplementary Figure 8 | User interface of the online version of REtarget. Screenshot of the web interface followed by detailed descriptions of each field in **Supplementary Note 1**.

Supplementary Table 1

Number of reads with max deletion length [bp] according to column name

Sample	***< 30***	***< 100***	***< 250***	***< 500***	***< 1000***	***< 2000***	***≥ 2000***
UROS background	179458	1	1	1	0	0	0
UROS A1	115173	934	432	611	773	137	83
UROS A1+B1	145966	1681	1455	1659	2344	390	208
UROS A1+B1+C1	139908	1513	1142	1141	1526	229	446
PLCB4 background	368958	7	0	0	0	0	0
PLCB4 A1	141850	3508	2237	2813	2978	771	291
PLCB4 A1+B1	129968	3507	2486	3106	3726	740	320
PLCB4 A1+B1+C1	82903	1877	1199	1413	1679	439	195
TRAC background	225241	1	1	1	1	0	0
TRAC A1	198693	2323	2062	2031	1699	76	27
TRAC A1+B1	190882	8105	6738	9723	6743	584	256

Percentage of reads with max deletion length [bp] according to column name

Sample	***< 30***	***< 100***	***< 250***	***< 500***	***< 1000***	***< 2000***	***≥ 2000***
UROS background	100.00	0.00	0.00	0.00	0.00	0.00	0.00
UROS A1	97.49	0.79	0.37	0.52	0.65	0.12	0.07
UROS A1+B1	94.97	1.09	0.95	1.08	1.53	0.25	0.14
UROS A1+B1+C1	95.89	1.04	0.78	0.78	1.05	0.16	0.31
PLCB4 background	100.00	0.00	0.00	0.00	0.00	0.00	0.00
PLCB4 A1	91.84	2.27	1.45	1.82	1.93	0.50	0.19
PLCB4 A1+B1	90.35	2.44	1.73	2.16	2.59	0.51	0.22
PLCB4 A1+B1+C1	92.42	2.09	1.34	1.58	1.87	0.49	0.22
TRAC background	100.00	0.00	0.00	0.00	0.00	0.00	0.00
TRAC A1	96.03	1.12	1.00	0.98	0.82	0.04	0.01
TRAC A1+B1	85.59	3.63	3.02	4.36	3.02	0.26	0.11

Supplementary Table 2

Off target #	Sample	Chromosome:Start-end	Cutsite	Mismatches	DISCOVER score	Guide sequence	PAM	Forward primer	Reverse primer	Nested forward primer	Nested reverse primer
OT1	A1 only	chr8:21029773-21029792	21029775	7	4	GGGAGAAGGAGGGTTTTGCC	NAG	CCTCACAGCCTGTAGACCCTA	CATCTTCTGATGCCTTTGCTCAGT		
OT2	A1 only	chr3:32324909-32324928	32324925	4	3	AGGAAGAGCAGAGTTATGTT	NAG	ACAGTGCACAAGCTGCTGAT	TGTAATGACCCACCGGGGATG	CTGGCTGGGCCTGAGAATCA	CCAGAAGTGGGGAGCTGAGG
OT3	A1 only	chr12:50567182-50567201	50567198	5	3	GGAAGTTGCAAAGATATGTA	NAG	ATTGATCTGTATGTGCACTGACTT	TCCGCTGGCCCTATCTTGAG	GACACACTTTTCTGTTGTAAAATTTCCATT	AATAAAATCTGTGGATTGCACCAATGTCAA
OT4	B1 only	chr4:83836323-83836342	83836325	7	5	AGTCAAGCAGAGTTGTTTTT	NGG	TTGGCTTCTGGGAATGTTGTGT	TCCAAGGGCATACATTTGGTTGA	TCTGTTTAATAGCATATAGGTATGCATCAA	ATCATAAAACTAATATCTGTTAAAACAAGTTAACT
OT5	B1 only	chr12:84866287-84866306	84866289	6	4	GAAGCACCAGAGTTCTCTCA	NGG	GGGCTGTAATGAGCTTGGTGA	ATTTATAAGAGGAGTGCATGTGTCA		
OT6	C1 only	chr1:71967420-71967439	71967436	7	6	TATCACCAGAGGCTTTTGTG	NGG	GCAATTCAGCCCAACCGTCAT	TTGTGTGTACGTGTTTCTTGGGA		
OT7	A1+B1+C1	chr1:205373338-205373357	205373340	7	5	CAAAGCAGGTGAGTGATGAG	NAG	TGACGCCCACAAACGAGAAC	ATCCAGGGCTTCCTTGCCG		
OT8	A1+B1+C1	chr11:95328154-95328173	95328156	7	5	GAAAGGAGCAGAGAAAGGCA	NAG	ACGAGGTTAAGGGGTGAAAGCA	AAGTCGGAACCCAGGAAAGGT		
OT9	A1+B1+C1	chr1:173824140-173824159	173824142	7	5	GAGGGAGCGGAGGTCTTCCT	NGG	CCAAATTCCCTCTCGCTCCAC	ACTGATACGGAATCGGCGAGG		
OT10	A1+B1+C1	chr7:99486720-99486739	99486736	7	5	GAAGCAGGGCAGATATGATG	NGG	TGTGGACAGTTGCAGGCTTACT	TGAGAATTCGCTCATGCCCCA		
OT11	A1+B1+C1	chrX:111305805-111305824	111305821	7	5	AAAGAAACAGAGCTATGCTA	NGG	GAAGTTTGCAACATAATGTTATGGGATAC	TTCGTTCATTACTTGATGCTTGCC	CTGTGTCTGGCTTATTTCAAAAGACA	TGTAATCCTCAGGGCAACCACT
OT12	A1+B1+C1	chr1:200620343-200620362	200620345	7	5	AATCACCGGATGTTTTGGTT	NGG	TCTGTTTGAGGAGTTTCGCCA	GAGTCTCCCCGAGTCGGTTG		
OT13	A1+B1+C1	chr6:147490336-147490355	147490352	6	4	AAAGCAGAAGAGATAGTTTA	NGG	TGAACAGAGGGTTGAAGAATCAG	TCTTCCAGCCCCACTTCAGA		
OT14	A1+B1+C1	chr7:67361251-67361270	67361253	4	3	ATGAAGCAGCGTTATTTGTC	NGG	CCACTCTTAACCTCACTCCTTGTG	TTGGAACTGAGAGTCCCCTCCC		
OT15	A1+B1+C1	chr1:112696607-112696626	112696609	5	3	GGAAGCAGAAGAGGTGTGGG	NAG	ACGGTGGGAATGAGTGGAGC	TTCTGGGTCAGTGGGGAACG	AGCCTGAGAGTCTGGTAGCTATAGCAG	GAACGCAGCACAGGCCCCAG
OT16	A1+B1+C1	chr3:14636237-14636256	14636239	5	3	GCAGCAGCAGATTCAGTGTC	NGG	CCTGCGGATGGCAGCTAGAA	TCATCCCTCTGTGTGTGCCAT	GTGGGGCCCTTGGGAGGTGATT	TGAGCTGGCTAAGACCTCTGGT

Supplementary Table 3

Locus	A1+ntc (%HDR)	A1+B1 (%HDR)	A1+B1+C1 (%HDR)	Absolute % change (A1 to B1)	Edit type	Cell type
UROS	35.33	51.32		15.99	3 bp ins	HEK-293T
UROS	23.36	48.40	62.69	25.04	3 bp ins	K-562
UROS	18.60	31.88		13.28	3 bp ins	HCT-116
UROS	3.50	12.00		8.50	3 bp ins	CD4+ T cell
PPP1R12C site 1 (gA1)	13.50	35.00		21.50	3 bp ins	HEK-293T
PPP1R12C site 1 (gA1)	20.00	35.50		15.50	3 bp ins	K-562
PPP1R12C site 1 (gA2)	21.00	39.50		18.50	3 bp ins	HEK-293T
PPP1R12C site 1 (gA2)	32.50	42.50		10.00	3 bp ins	K-562
PLCB4	16.50	31.00	48.00	14.50	3 bp ins	HEK-293T
GOLGA3	6.09	12.52		6.43	3 bp ins	HEK-293T
FAM134B	28.97	41.76		12.78	3 bp ins	HEK-293T
RP11-280G9.1	29.27	39.63		10.36	3 bp ins	HEK-293T
MFGE8	11.37	17.31		5.93	3 bp ins	HEK-293T
KCNQ4	24.75	33.70		8.95	3 bp ins	HEK-293T
ABCA3	36.32	59.06		22.73	2 bp del	HEK-293T
TEX45	45.73	59.73		14.00	3 bp ins	HEK-293T
TEX45	12.21	15.89		3.68	3 bp ins	K-562
PPP1R12C site 2	33.88	36.90		3.02	3 bp ins	HEK-293T
PPP1R12C site 2	20.72	34.41		13.69	3 bp ins	K-562
DACT2	9.98	13.43		3.45	3 bp ins	HEK-293T
ANK1	6.26	8.16		1.90	3 bp ins	HEK-293T
ZEB1	2.64	3.69		1.05	3 bp ins	HEK-293T
RASAL2	7.87	11.19		3.32	3 bp ins	HEK-293T
PAQR5	4.09	6.88		2.79	3 bp ins	HEK-293T
SLX4	4.05	5.56		1.51	3 bp ins	HEK-293T
LARGE	19.01	20.82		1.81	3 bp ins	HEK-293T
HIST1H2BJ	2.77	10.28		7.51	GFP	K-562
RAB11A	4.86	11.35		6.50	GFP	K-562
FBL	4.37	9.23		4.86	GFP	K-562
PPP1R12C site 1 (gA1)	0.17	0.68		0.52	GFP	K-562
TRAC	0.46	0.84		0.38	CAR	CD4+ T cell

Supplementary Table 4

Name	Sequence (5'-to-3')	Target
UROS gA1	GGAAGCAGCAGAGTTATGTT	wt
UROS gB1	GAAGCAGCAGAGTTATTGTT	1 bp ins
UROS gB2	GCAGTGGAAGCAGCAGAGTT	5 bp del
UROS gC1	AAGCAGCAGAGTTATTTGTT	2 bp ins
TEX45 gA1	GCGCGTCCTCGTGCAGGTGC	wt
TEX45 gB1	CGCGTCCTCGTGCAGGTTGC	6 bp del
PPP1R12C site 1 gA1	TCCCTAGTGGCCCCACTGTG	wt
PPP1R12C site 1 gA2	GTCCCCTCCACCCCACAGTG	wt
PPP1R12C site 1 gB1	CCCTAGTGGCCCCACTTGTG	1 bp ins
PPP1R12C site 2 gA1	TGGGGGTTAGACCCAATATC	wt
PPP1R12C site 2 gB1	GGGGGTTAGACCCAATTATC	1 bp ins
PPP1R12C site 2 gB2	GGTGGGGGTTAGACCCAATC	2 bp del
PLCB4 gA1	CGCTATACCACCACCACCAC	wt
PLCB4 gB1	CACCGCTATACCACCACCAC	3 bp del
PLCB4 gC1	ACTCACCGCTATACCACCAC	6 bp del
ANK1 gA1	ATCTGAGCTCCTATGCTGCA	wt
ANK1 gB1	TCTGAGCTCCTATGCTTGCA	1 bp ins
SLX4 gA1	CCGTGCAAACTCCTGCTGCA	wt
SLX4 gB1	CGTGCAAACTCCTGCTTGCA	1 bp ins
DACT2 gA1	CGCAGCTTGCTCCTGCAGCA	wt
DACT2 gB1	GCAGCTTGCTCCTGCAAGCA	1 bp ins
ZEB1 gA1	GCTTCTCAGCTTCTGCTGCA	wt
ZEB1 gB1	CTTCTCAGCTTCTGCTTGCA	1 bp ins
GOLGA3 gA1	CTCCTTAGCGCAGCACAGCA	wt
GOLGA3 gB1	TCCTTAGCGCAGCACAAGCA	3 bp del
MFGE8 gA1	TCTAACTGCCAGCTGCTGGA	wt
MFGE8 gB1	ATCCCTGCCCTCCAAGCAGC	1 bp ins
FAM134B gA1	ATTCCACCTTCTACCTGACC	wt
FAM134B gB1	CTCCTATTCCACCTTCTACC	5 bp del
RP11-280G9.1 gA1	AGGCTCAAGACTCCTCACTC	wt
RP11-280G9.1 gB1	GCTCAAGACTCCTCAGGAGA	5 bp del
RASAL2 gA1	ACTCACTAACACCACTGCAC	wt
RASAL2 gB1	TGTATACTCACTAACACCAC	4 bp del
PAQR5 gA1	TTCTGCTTGTCACAGCAGCG	wt
PAQR5 gB1	TCTGCTTGTCACAGCAAGCG	1 bp ins
KCNQ4 gA1	GACCATTATCCTCAGCAGCG	wt
KCNQ4 gB1	ACCATTATCCTCAGCAAGCG	1 bp ins
ABCA3 gA1	AACCTGCTTCAGAGACTCAG	wt
ABCA3 gB1	ACCTGCTTCAGAGACTTCAG	1 bp ins
LARGE gA1	GACTAGCCAAAGCTGAGAGC	wt
LARGE gB1	ACCCAGGGACTAGCCAAAGC	7 bp del
HIST1H2BJ gA1	GCGCTAAGTAAACAGTGAGT	wt
HIST1H2BJ gB1	ACCAGCGCTAAGTAAACAGT	4 bp del
FBL gA1	AACTGAAGTTCAGCGCTGTC	wt
FBL gB1	ACTGAAGTTCAGCGCTTGTC	1 bp ins
RAB11A gA1	GGTAGTCGTACTCGTCGTCG	wt
RAB11A gB1	GAGGTAGTCGTACTCGTCGC	3 bp del
TRAC gA1	AGAGTCTCTCAGCTGGTACA	wt
TRAC gB1	GAGTCTCTCAGCTGGTTACA	1 bp ins
TRAC (g526)	TCAGGGTTCTGGATATCTGT	wt
Promiscuous gRNA	GCCATGAATTCATAGGGAAT	wt (14x sites)
Non-targeting control (ntc)	GATATCCCGGTGGGGTTCTC	-

Supplementary Table 5

Name	Sequence (5'-to-3')
UROS	CGGGGACTCATTTTTACCAGCCCCAGAGCAGTGGAAGCAGCAGAGTTAT GAT GTTTGGAGCAAAACAATAAAACTGAAGGTGAGGGTGGGTCTGCTGTGGAT
TEX45	CGGCGTGCGCGTTGGAGAGGCCGATCCCGGCGTGCGCGTCCTCGTGCAGG GAT TGCAGGTTGCCGGCCTGCATGGCGAGCGTGCGTTCCCGCGCCTGCGCTTG
PPP1R12C site 1	CTCTGGTTCTGGGTACTTTTATCTGTCCCCTCCACCCCAC GAT AGTGGGGCCACTAGGGACAGGATTGGTGACAGAAAAGCCC
PPP1R12C site 2	AAGCCCCATCCTTAGGCCTCCTCCTTCCTAGTCTCCTGAT GAT ATTGGGTCTAACCCCCACCTCCTGTTAGGCAGATTCCTTA
PLCB4	GTTTGGACGGCAGCTGTACTCACCGCTATACCACCACCAC GAT CACTGGCTGGGCTTCTCAGTTTTAAACTCTAGGCTTTCAG
ANK1	ATTGTTTACCAGGACTGGGGTCAGCCTTCCACACCCCTGC GAT AGCATAGGAGCTCAGATCACTTTCTCTGGGAAGGTGGGGA
SLX4	GCTTGGGCCCCCTGCCCCAGAGAGCGACGCTGCGGTGGCCTTGACCCTGC GAT AGCAGGAGTTTGCACGGGTAGGAGCATCGGCACATGATGATAGCCTGGAG
DACT2	TGGCAGCTTGCAACAATTCCTTTTCCCTGGCAGCTTCCCACATTCCATGC GAT TGCAGGAGCAAGCTGCGCGTCTGATTCTGCCGCATCAACATGATGCCTGG
GOLGA3	GGTCCAGCAGGGGCCCTGCCCACCAGAACACAGTCCGTGC GAT TGTGCTGCGCTAAGGAGCTGGCCCTCAACTCTCCTTGGTG
MFGE8	GATGTTCCTGTGCCCCAGGAGTTGTCTGATTTCTCTAACTGCCAGCTGCT GAT GGAGGGCAGGGATGTTGTCCTGTTCATCACTGGGTCGTCACTAGGCAGGC
FAM134B	CTCACCCGCCCTGCCTCGAAGTCCCCTTCCCTGCTGTGACCAGGT GAT CAGGTAGAAGGTGGAATAGGAGTGGGCAGGTATTCAATTTATACA
RP11-280G9.1	CTGAGTGGAAGGCTCAGAGGCCCCAAGGTAGCACCCTCTCCTGAG GAT TGAGGAGTCTTGAGCCTCAGCAGCAAGGGGGAATTTAGTGAATCT
RASAL2	GTGGTTCACAAGTAGAAGGGAAAAGAGAAGTCTTGAGGCCCAGTG GAT CAGTGGTGTTAGTGAGTATACAGTAAACAAAAGAAATAAAAATTG
PAQR5	TCAACCCTGGCAAAAGTCATCCTCTTCTCTCTCTTCTGCTTGTCACAGCA GAT GCGGGGGAACTTCGCTCCCATCTGAAGTGAGCCTCAGCCCTCTTGGTGCC
KCNQ4	GAGTGCCTGTGATGTTCCAGGTCTGAAGAGCCATCCTCGC GAT TGCTGAGGATAATGGTCCTGTGCTCCCATGGGACCCCTCT
LARGE	ATCAGCAAGGGATGCTGGGGCACCCAGGGACTAGCCAAAGCTGAG GAT AGCTGGTACCAGTTCTGGGCTGAGAGATGTGTGTTAGTTTGCTAG
ABCA3 (Arg1333fs)	CCTGCTCTTCCTCATCGAGACCAACCTGCTTCAGAGACTCGGGCATCCTCTGCGCCCTCCGGAGGAGGCGGACACTGGTG
ZEB1 (Glu566X)	ATGACCTAAAGCAGCCTACTCAGCCTCCTCCACTaCCTGC AGCAtAAGCTGAGAAGCCTGAGTCCTCTGTTTCATCAGCT
HIST1H2BJ-GFP (Addgene #109121)	AAGGTTCTGAAGCAGGTCCACCCTGACACCGGCATTTCGTCCAAGGCCATGGGCATCATGAATTCGTTTGTGAACGACATTTTCGAGCGCATCGCAGGTGAGGCTTCCCGCCTGGCGCATTACAACAAGCGCTCGACCATCACCTCCAGGGAGATCCAGACGGCCGTGCGCCTGCTGCTGCCTGGGGAGTTGGCCAAGCACGCCGTGTCCGAGGGTAC<br>TAAGGCCGTCACCAAGTACACCAGCGCTAAGGACCCTCCTGTGGCCACCGTGAGCAAGGGCGAGGAGCTGTTCACCGGGGTGGTGCCCATCCTGGTCGAGCTGGACGGCGACGTAAACGGCCACAAGTTCAGCGTGTCCGGCGAGGGCGAGGGCGATGCCACCTACGGCAAGCTGACCCTGAAGTTCATCTGCACCACCGGCAAGCTGCCCGTGCCCT<br>GGCCCACCCTCGTGACCACCCTGACCTACGGCGTGCAGTGCTTCAGCCGCTACCCCGACCACATGAAGCAGCACGACTTCTTCAAGTCCGCCATGCCCGAAGGCTACGTCCAGGAGCGCACCATCTTCTTCAAGGACGACGGCAACTACAAGACCCGCGCCGAGGTGAAGTTCGAGGGCGACACCCTGGTGAACCGCATCGAGCTGAAGGGCATCGAC<br>TTCAAGGAGGACGGCAACATCCTGGGGCACAAGCTGGAGTACAACTACAACAGCCACAACGTCTATATCATGGCCGACAAGCAGAAGAACGGCATCAAGGTGAACTTCAAGATCCGCCACAACATCGAGGACGGCAGCGTGCAGCTCGCCGACCACTACCAGCAGAACACCCCCATCGGCGACGGCCCCGTGCTGCTGCCCGACAACCACTACCTGAG<br>CACCCAGTCCAAGCTGAGCAAAGACCCCAACGAGAAGCGCGATCACATGGTCCTGCTGGAGTTCGTGACCGCCGCCGGGATCACTCTCGGCATGGACGAGCTGTACAAGTAAACAGTGAGTTGGTTGCAAACTCTCAATCCTAACGCCTCTTTTAAGAGCCACCCATGTTCTCAAAGAAAGAGCTGGTGCTTGTATTCCTCCTCTGCTGGCCACTGAC<br>AAACCCTTGTAACTTGCTACTGTGTTTTTGGTCTGAAGTAGAGCAGTTATTTAACTAATCCTTAGTGACTTTTTTTTTTTAGATCTGCCATTCTAATCTTAGAGTTAAGTAAGGAGATGGGAAATTTTCTATTATAAGTTCGAAACCAATTAAAATACGTTAGAAACCAATTAAAATACTCGTCGGTCCCCCGTCGGTTAGTGATTTGGAACAGTGC<br>CAAGTTGCAGCGGTTGTCAGTTTGAATTTGCCCG
FBL-GFP (Addgene #87427)	GAAAAACAGCCCAAAGCCCTGTTGTAGACATTAGTCCTTTCTCCTCTTTAGGCCAACTGCATTGACTCCACAGCCTCAGCCGAGGCCGTGTTTGCCTCCGAAGTGAAAAAGATGCAACAGGAGAACATGAAGCCGCAGGAGCAGTTGACCCTTGAGCCATATGAAAGAGACCATGCCGTGGTCGTGGGAGTGTACAGGTGAGCAGGGGCCCAGCAATA<br>CACCAAGACAGACATCTCTGTCCCTTGCACCCCGAGTGCCATGATCCTGGGGACCCTCCTTCATCACCTATCTTCCTCTCACAGGCCACCTCCTAAAGTGAAGAACAAGCCCAACAGCGCCGTGGACGGCACCGCCGGCCCGGCGTGAGCAAGGGCGAGGAGCTGTTCACCGGGGTGGTGCCCATCCTGGTCGAGCTGGACGGCGACGTAAACGGCC<br>ACAAGTTCAGCGTGTCCGGCGAGGGCGAGGGCGATGCCACCTACGGCAAGCTGACCCTGAAGTTCATCTGCACCACCGGCAAGCTGCCCGTGCCCTGGCCCACCCTCGTGACCACCCTGACCTACGGCGTGCAGTGCTTCAGCCGCTACCCCGACCACATGAAGCAGCACGACTTCTTCAAGTCCGCCATGCCCGAAGGCTACGTCCAGGAGCGCACC<br>ATCTTCTTCAAGGACGACGGCAACTACAAGACCCGCGCCGAGGTGAAGTTCGAGGGCGACACCCTGGTGAACCGCATCGAGCTGAAGGGCATCGACTTCAAGGAGGACGGCAACATCCTGGGGCACAAGCTGGAGTACAACTACAACAGCCACAACGTCTATATCATGGCCGACAAGCAGAAGAACGGCATCAAGGTGAACTTCAAGATCCGCCACAA<br>CATCGAGGACGGCAGCGTGCAGCTCGCCGACCACTACCAGCAGAACACCCCCATCGGCGACGGCCCCGTGCTGCTGCCCGACAACCACTACCTGAGCACCCAGTCCAAGCTGAGCAAAGACCCCAACGAGAAGCGCGATCACATGGTCCTGCTGGAGTTCGTGACCGCCGCCGGGATCACTCTCGGCATGGACGAGCTGTACAAGTAATGAAGTTCAG<br>CCCTGAGCGGATTGCGAGAGATGTGTGTTGATACTGTTGCACGTGTGTTTTTCTATTAAAAGACTCATCCGTCTCCCATGTCTGCTGCTCATTCCTCCCCTTGACCTGCTGACACAGGGAGCACGCACCCTTGGTCAATTTTGCGGGGTTGGGTAAATTCTCACTCGGTCACAGAGCGCATGCTCCGTTTCTAGCTGCCTTTGCGCAGCGGCAGCCTG<br>GATTTCGGTTCTTGGGTGGGATTGGTAGCTCGCTGCGCATGCGTGCAGGTAAGCGGCCATCTCGCGCAGGCGGAGTGTCAGTGTGGGTCACGTGAGGGGAGCGGAGAGGGAGGGATGGGGGCGGAGTCCAGGGCGTGGGGGGGCCGGTTTGTTGTGGTCGCCATTTTGCT
RAB11A-GFP (Addgene #112012)	GGTAGCTAGGAGTTCCAGGACTCAGTTTCCCCTTTGAGCCTCCTTTAGCGACTAAAGCTTGAAGCCCCACGCATCTCGACTCTCGCGCACACCGCCCTTGTTGGGCTCAGGGGCGGGGCGCCGCCCCCGGAAGTACTTCCCCTTAAAGGCTGGGGCCTGCCGGAAATGGCGCAGCGGCAGGGAGGGGCTCTTCACCCAGTCCGGCAGTTGAAGCTCGG<br>CGCTCGGGTTACCCCTGCAGCGACGCCCCCTGGTCCCACAGATACCACTGCTGCTCCCGCCCTTTCGCTCCTCGGCCGCGCAATGGGCGGATCGGGTGGGACTAGTGGCAGCAAGGGCGAGGAGCTGTTCACCGGGGTGGTGCCCATCCTGGTCGAGCTGGACGGCGACGTAAACGGCCACAAGTTCAGCGTGCGCGGCGAGGGCGAGGGCGATGCCA<br>CCAACGGCAAGCTGACCCTGAAGTTCATCTGCACCACCGGCAAGCTGCCCGTGCCCTGGCCCACCCTCGTGACCACCCTGACCTACGGCGTGCAGTGCTTCAGCCGCTACCCCGACCACATGAAGCGCCACGACTTCTTCAAGTCCGCCATGCCCGAAGGCTACGTCCAGGAGCGCACCATCAGCTTCAAGGACGACGGCACCTACAAGACCCGCGCC<br>GAGGTGAAGTTCGAGGGCGACACCCTGGTGAACCGCATCGAGCTGAAGGGCATCGACTTCAAGGAGGACGGCAACATCCTGGGGCACAAGCTGGAGTACAACTTCAACAGCCACAACGTCTATATCACCGCCGACAAGCAGAAGAACGGCATCAAGGCCAACTTCAAGATCCGCCACAACGTGGAGGACGGCAGCGTGCAGCTCGCCGACCACTACCA<br>GCAGAACACCCCCATCGGCGACGGCCCCGTGCTGCTGCCCGACAACCACTACCTGAGCACCCAGTCCGTGCTGAGCAAAGACCCCAACGAGAAGCGCGATCACATGGTCCTGCTGGAGTTCGTGACCGCCGCCGGGATCACTGGAACCGGTGCTGGAAGTGGTACACGCGACGACGAGTACGACTACCTCTTTAAAGGTGAGGCCATGGGCTCTCGCA<br>CTCTACACAGTCCTCGTTCGGGGACCCGGGCCACTCCCGGTGGACCCTCGTGCCGGCCACCCCTGCACTGATATAGGCCTCCCTCAGCCCTTCCTTTTTGTGCGGTTCCGTCTCCTACCCAGCTCAGCCTCTTCTCCCCCGCTCAGACAGGGGTCCCCATCACATGCCGCTCTCTGAGCGACCTCTCCATAGGCCTTCGCTGGCCTCAGAGCCCCTCC<br>CTGCGTGTCCTTCCCCTGGCGGACTGCCTTCTCCCACATCGT
AAVS1-GFP (Addgene #52344)	TGCTTTCTCTGACCAGCATTCTCTCCCCTGGGCCTGTGCCGCTTTCTGTCTGCAGCTTGTGGCCTGGGTCACCTCTACGGCTGGCCCAGATCCTTCCCTGCCGCCTCCTTCAGGTTCCGTCTTCCTCCACTCCCTCTTCCCCTTGCTCTCTGCTGTGTTGCTGCCCAAGGATGCTCTTTCCGGAGCACTTCCTTCTCGGCGCTGCACCACGTGATGTC<br>CTCTGAGCGGATCCTCCCCGTGTCTGGGTCCTCTCCGGGCATCTCTCCTCCCTCACCCAACCCCATGCCGTCTTCACTCGCTGGGTTCCCTTTTCCTTCTCCTTCTGGGGCCTGTGCCATCTCTCGTTTCTTAGGATGGCCTTCTCCGACGGATGTCTCCCTTGCGTCCCGCCTCCCCTTCTTGTAGGCCTGCATCATCACCGTTTTTCTGGACAACC<br>CCAAAGTACCCCGTCTCCCTGGCTTTAGCCACCTCTCCATCCTCTTGCTTTCTTTGCCTGGACACCCCGTTCTCCTGTGGATTCGGGTCACCTCTCACTCCTTTCATTTGGGCAGCTCCCCTACCCCCCTTACCTCTCTAGTCTGTGCTAGCTCTTCCAGCCCCCTGTCATGGCATCTTCCAGGGGTCCGAGAGCTCAGCTAGTCTTCTTCCTCCAAC<br>CCGGGCCCCTATGTCCACTTCAGGACAGCATGTTTGCTGCCTCCAGGGATCCTGTGTCCCCGAGCTGGGACCACCTTATATTCCCAGGGCCGGTTAATGTGGCTCTGGTTCTGGGTACTTTTATCTGTCCCCTCCACCCCACAGTGGGGCAAGCTTCTGACCTCTTCTCTTCCTCCCACAGGGCCTCGAGAGATCTGGCAGCGGAGAGGGCAGAGGAA<br>GTCTTCTAACATGCGGTGACGTGGAGGAGAATCCCGGCCCTAGGCTCGAGATGACCGAGTACAAGCCCACGGTGCGCCTCGCCACCCGCGACGACGTCCCCAGGGCCGTACGCACCCTCGCCGCCGCGTTCGCCGACTACCCCGCCACGCGCCACACCGTCGATCCGGACCGCCACATCGAGCGGGTCACCGAGCTGCAAGAACTCTTCCTCACGCGC<br>GTCGGGCTCGACATCGGCAAGGTGTGGGTCGCGGACGACGGCGCCGCGGTGGCGGTCTGGACCACGCCGGAGAGCGTCGAAGCGGGGGCGGTGTTCGCCGAGATCGGCCCGCGCATGGCCGAGTTGAGCGGTTCCCGGCTGGCCGCGCAGCAACAGATGGAAGGCCTCCTGGCGCCGCACCGGCCCAAGGAGCCCGCGTGGTTCCTGGCCACCGTCGG<br>CGTCTCGCCCGACCACCAGGGCAAGGGTCTGGGCAGCGCCGTCGTGCTCCCCGGAGTGGAGGCGGCCGAGCGCGCCGGGGTGCCCGCCTTCCTGGAGACCTCCGCGCCCCGCAACCTCCCCTTCTACGAGCGGCTCGGCTTCACCGTCACCGCCGACGTCGAGGTGCCCGAAGGACCGCGCACCTGGTGCATGACCCGCAAGCCCGGTGCCTGATCTA<br>GAGGGCCCGTTTAAACCCGCTGATCAGCCTCGACTGTGCCTTCTAGTTGCCAGCCATCTGTTGTTTGCCCCTCCCCCGTGCCTTCCTTGACCCTGGAAGGTGCCACTCCCACTGTCCTTTCCTAATAAAATGAGGAAATTGCATCGCATTGTCTGAGTAGGTGTCATTCTATTCTGGGGGGTGGGGTGGGGCAGGACAGCAAGGGGGAGGATTGGGAA<br>GACAATAGCAGGCATGCTGGGGATGCGGTGGGCTCTATGGGTCTCGACATTGATTATTGACTAGTTATTAATAGTAATCAATTACGGGGTCATTAGTTCATAGCCCATATATGGAGTTCCGCGTTACATAACTTACGGTAAATGGCCCGCCTGGCTGACCGCCCAACGACCCCCGCCCATTGACGTCAATAATGACGTATGTTCCCATAGTAACGCCA<br>ATAGGGACTTTCCATTGACGTCAATGGGTGGACTATTTACGGTAAACTGCCCACTTGGCAGTACATCAAGTGTATCATATGCCAAGTACGCCCCCTATTGACGTCAATGACGGTAAATGGCCCGCCTGGCATTATGCCCAGTACATGACCTTATGGGACTTTCCTACTTGGCAGTACATCTACGTATTAGTCATCGCTATTACCATGGTCGAGGTGA<br>GCCCCACGTTCTGCTTCACTCTCCCCATCTCCCCCCCCTCCCCACCCCCAATTTTGTATTTATTTATTTTTTAATTATTTTGTGCAGCGATGGGGGCGGGGGGGGGGGGGGGCGCGCGCCAGGCGGGGCGGGGCGGGGCGAGGGGCGGGGCGGGGCGAGGCGGAGAGGTGCGGCGGCAGCCAATCAGAGCGGCGCGCTCCGAAAGTTTCCTTTTATGGC<br>GAGGCGGCGGCGGCGGCGGCCCTATAAAAAGCGAAGCGCGCGGCGGGCGGGAGTCGCTGCGTTGCCTTCGCCCCGTGCCCCGCTCCGCGCCGCCTCGCGCCGCCCGCCCCGGCTCTGACTGACCGCGTTACTCCCACAGGTGAGCGGGCGGGACGGCCCTTCTCCTCCGGGCTGTAATTAGCGCTTGGTTTAATGACGGCTCGTTTCTTTTCTGTGGC<br>TGCGTGAAAGCCTTAAAGGGCTCCGGGAGGGCCCTTTGTGCGGGGGGGAGCGGCTCGGGGGGTGCGTGCGTGTGTGTGTGCGTGGGGAGCGCCGCGTGCGGCCCGCGCTGCCCGGCGGCTGTGAGCGCTGCGGGCGCGGCGCGGGGCTTTGTGCGCTCCGCGTGTGCGCGAGGGGAGCGCGGCCGGGGGCGGTGCCCCGCGGTGCGGGGGGGCTGCGA<br>GGGGAACAAAGGCTGCGTGCGGGGTGTGTGCGTGGGGGGGTGAGCAGGGGGTGTGGGCGCGGCGGTCGGGCTGTAACCCCCCCCTGCACCCCCCTCCCCGAGTTGCTGAGCACGGCCCGGCTTCGGGTGCGGGGCTCCGTGCGGGGCGTGGCGCGGGGCTCGCCGTGCCGGGCGGGGGGTGGCGGCAGGTGGGGGTGCCGGGCGGGGCGGGGCCGCCT<br>CGGGCCCGGGGAGGGCTCGGGGGAGGGGCGCGGCGGCCCCGGAGCGCCGGCGGCTGTCGAGGCGCGGCGAGCCGCAGCCATTGCCTTTTATGGTAATCGTGCGAGAGGGCGCAGGGACTTCCTTTGTCCCAAATCTGGCGGAGCCGAAATCTGGGAGGCGCCGCCGCACCCCCTCTAGCGGGCGCGGGGCGAAGCGGTGCGGCGCCGGCAGGAAGGAAAT<br>GGGCGGGGAGGGCCTTCGTGCGTCGCCGCGCCGCCGTCCCCTTCTCCATCTCCAGCCTCGGGGCTGCCGCAGGGGGACGGCTGCCTTCGGGGGGGACGGGGCAGGGCGGGGTTCGGCTTCTGGCGTGTGACCGGCGGCTCTAGAGCCTCTGCTAACCATGTTCATGCCTTCTTCTTTTTCCTACAGCTCCTGGGCAACGTGCTGGTTGTTGTGCTGTC<br>TCATCATTTTGGCAAAGAATTCGTCGACCACCATGGTGAGCAAGCAGATCCTGAAGAACACCGGCCTGCAGGAGATCATGAGCTTCAAGGTGAACCTGGAGGGCGTGGTGAACAACCACGTGTTCACCATGGAGGGCTGCGGCAAGGGCAACATCCTGTTCGGCAACCAGCTGGTGCAGATCCGCGTGACCAAGGGCGCCCCCCTGCCCTTCGCCTTC<br>GACATCCTGAGCCCCGCCTTCCAGTACGGCAACCGCACCTTCACCAAGTACCCCGAGGACATCAGCGACTTCTTCATCCAGAGCTTCCCCGCCGGCTTCGTGTACGAGCGCACCCTGCGCTACGAGGACGGCGGCCTGGTGGAGATCCGCAGCGACATCAACCTGATCGAGGGGATGTTCGTGTACCGCGTGGAGTACAAGGGCCGCAACTTCCCCAA<br>CGACGGCCCCGTGATGAAGAAGACCATCACCGGCCTGCAGCCCAGCTTCGAGGTGGTGTACATGAACGACGGCGTGCTGGTGGGCCAGGTGATCCTGGTGTACCGCCTGAACAGCGGCAAGTTCTACAGCTGCCACATGCGCACCCTGATGAAGAGCAAGGGCGTGGTGAAGGACTTCCCCGAGTACCACTTCATCCAGCACCGCCTGGAGAAGACCT<br>ACGTGGAGGACGGCGGCTTCGTAGAGCAGCACGAGACCGCCATCGCCCAGCTGACCAGCCTGGGCAAGCCCCTGGGCAGCCTGCACGAGTGGGTGTAAATCGATGATATCACGCGTGAATTCACTCCTCAGGTGCAGGCTGCCTATCAGAAGGTGGTGGCTGGTGTGGCCAATGCCCTGGCTCACAAATACCACTGAGATCTTTTTCCCTCTGCCAAA<br>AATTATGGGGACATCATGAAGCCCCTTGAGCATCTGACTTCTGGCTAATAAAGGAAATTTATTTTCATTGCAATAGTGTGTTGGAATTTTTTGTGTCTCTCACTCGGAAGGACATATGGGAGGGCAAATCATTTAAAACATCAGAATGAGTATTTGGTTTAGAGTTTGGCAACATATGCCCATATGCTGGCTGCCATGAACAAAGGTTGGCTATAAAG<br>AGGTCATCAGTATATGAAACAGCCCCCTGCTGTCCATTCCTTATTCCATAGAAAAGCCTTGACTTGAGGTTAGATTTTTTTTATATTTTGTTTTGTGTTATTTTTTTCTTTAACATCCCTAAAATTTTCCTTACATGTTTTACTAGCCAGATTTTTCCTCCTCTCCTGACTACTCCCAGTCATAGCTGTCCCTCTTCTCTTATGGAGATCCCTCGACC<br>TGCAGCCCAAGCTTGGCGTAATCATGGTCATAGCTGTTTCCTGTGTGAAATTGTTATCCGCTCACAATTCCACACAACATACGAGCCGGAAGCATAAAGTGTAAAGCCTGGGGTGCCTAATGAGTGAGCTAACTCACATTAATTGCGTTGCGCTCACTGCCCGCTTTCCAGTCGGGAAACCTGTCGTGCCAGCGGATCGACAGTACTAAGCTTTACTA<br>GGGACAGGATTGGTGACAGAAAAGCCCCATCCTTAGGCCTCCTCCTTCCTAGTCTCCTGATATTGGGTCTAACCCCCACCTCCTGTTAGGCAGATTCCTTATCTGGTGACACACCCCCATTTCCTGGAGCCATCTCTCTCCTTGCCAGAACCTCTAAGGTTTGCTTACGATGGAGCCAGAGAGGATCCTGGGAGGGAGAGCTTGGCAGGGGGTGGGAG<br>GGAAGGGGGGGATGCGTGACCTGCCCGGTTCTCAGTGGCCACCCTGCGCTACCCTCTCCCAGAACCTGAGCTGCTCTGACGCGGCTGTCTGGTGCGTTTCACTGATCCTGGTGCTGCAGCTTCCTTACACTTCCCAAGAGGAGAAGCAGTTTGGAAAAACAAAATCAGAATAAGTTGGTCCTGAGTTCTAACTTTGGCTCTTCACCTTTCTAGTCCCC<br>AATTTATATTGTTCCTCCGTGCGTCAGTTTTACCTGTGAGATAAGGCCAGTAGCCAGCCCCGTCCTGGCAGGGCTGTGGTGAGGAGGGGGGTGTCCGTGTGGAAAACTCCCTTTGTGAGAATGGTGCGTCCTAGGTGTTCACCAGGTCGTGGCCGCCTCTACTCCCTTTCTCTTTCTCCATCCTTCTTTCCTTAAAGAGTCCCCAGTGCTATCTGGGA<br>CATATTCCTCCGCCCAGAGCAGGGTCCCGCTTCCCTAAGGCCCTGCTCTGGGCTTCTGGGTTTGAGTCCTTGGCAAGCCCAGGAGAGGCGCTCAGGCTTCCCTGTCCCCCTTCCTCGTCCACCATCTCATGCCCCTGGCTCTCCTGCCCCTTCCCTACAGGGGTTCCTGGCTCTGCTC
BCMA-CAR-myc	TTTCAGGTTTCCTTGAGTGGCAGGCCAGGCCTGGCCGTGAACGTTCACTGAAATCATGGCCTCTTGGCCAAGATTGATAGCTTGTGCCTGTCCCTGAGTCCCAGTCCATCACGAGCAGCTGGTTTCTAAGATGCTATTTCCCGTATAAAGCATGAGACCGTGACTTGCCAGCCCCACAGAGCCCCGCCCTTGTCCATCACTGGCATCTGGACTCCAGC<br>CTGGGTTGGGGCAAAGAGGGAAATGAGATCATGTCCTAACCCTGGAATTGGATCCTCTTGTCTTACAGATGGATCTGGAGCAACAAACTTCTCACTACTCAAACAAGCAGGTGACGTGGAGGAGAATCCCGGCCCCATGAAATGGAAAGCACTCTTTACCGCCGCAATCCTTCAAGCACAGTTGCCAATTACCGAGGCTGACATTGTACTGACGCAAA<br>GTCCCCCTAGCTTGGCGATGAGTCTCGGGAAGCGAGCGACGATTAGTTGCCGAGCTTCTGAAAGTGTCACAATCCTTGGCTCCCACCTGATCCATTGGTACCAACAAAAACCTGGGCAGCCCCCGACGCTTCTCATTCAGTTGGCGTCTAACGTGCAAACAGGAGTACCGGCCAGATTTTCAGGCTCAGGCTCTCGCACCGACTTTACTCTGACCATC<br>GACCCTGTTGAGGAGGATGATGTAGCAGTTTACTACTGTCTTCAGAGCAGAACCATTCCTCGCACATTCGGCGGTGGAACGAAGTTGGAAATCAAGGGCTCAACAAGTGGGAGTGGGAAGCCCGGCAGCGGGGAGGGTTCTACTAAAGGCCAAATACAGTTGGTTCAATCCGGGCCTGAACTGAAAAAGCCGGGAGAGACCGTGAAAATTTCTTGCAA<br>GGCTAGCGGGTACACTTTTACGGATTACTCTATTAACTGGGTTAAGAGGGCACCGGGCAAAGGGCTGAAATGGATGGGCTGGATAAACACCGAGACTCGGGAGCCTGCATATGCTTATGATTTCAGAGGTAGATTTGCGTTCTCTTTGGAAACCTCAGCTTCAACGGCCTACTTGCAGATTAATAACTTGAAGTACGAAGACACCGCCACTTACTTCT<br>GCGCTCTCGATTACTCATACGCTATGGATTACTGGGGCCAGGGCACGTCCGTGACCGTGTCCAGCGAACAGAAACTTATATCAGAGGAAGACCTTGCAATTGAAGTTATGTATCCTCCTCCTTACCTAGACAATGAGAAGAGCAATGGAACCATTATCCATGTGAAAGGGAAACACCTTTGTCCAAGTCCCCTATTTCCCGGACCTTCTAAGCCCTTT<br>TGGGTGCTGGTGGTGGTTGGTGGAGTCCTGGCTTGCTATAGCTTGCTAGTAACAGTGGCCTTTATTATTTTCTGGGTGAGGAGTAAGAGGAGCAGGCTCCTGCACAGTGACTACATGAACATGACTCCCCGCCGCCCCGGGCCCACCCGCAAGCATTACCAGCCCTATGCCCCACCACGCGACTTCGCAGCCTATCGCTCCAGAGTGAAGTTCAGCAG<br>GAGCGCAGACGCCCCCGCGTACCAGCAGGGCCAGAACCAGCTCTATAACGAGCTCAATCTAGGACGAAGAGAGGAGTACGATGTTTTGGACAAGAGACGTGGCCGGGACCCTGAGATGGGGGGAAAGCCGAGAAGGAAGAACCCTCAGGAAGGCCTGTACAATGAACTGCAGAAAGATAAGATGGCGGAGGCCTACAGTGAGATTGGGATGAAAGGCG<br>AGCGCCGGAGGGGCAAGGGGCACGATGGCCTTTACCAGGGTCTCAGTACAGCCACCAAGGACACCTACGACGCCCTTCACATGCAGGCCCTGCCCCCTCGCGGAAGCGGAGCTACTAACTTCAGCCTGCTGAAGCAGGCTGGAGACGTGGAGGAGAACCCTGGACCCAATATCCAGAACCCTGACCCTGCGGTGTACCAGCTGAGAGACTCTAAATCC<br>AGTGACAAGTCTGTCTGCCTATTCACCGATTTTGATTCTCAAACAAATGTGTCACAAAGTAAGGATTCTGATGTGTATATCACAGACAAAACTGTGCTAGACATGAGGTCTATGGACTTCAAGAGCAACAGTGCTGTGGCCTGGAGCAACAAATCTGACTTTGCATGTGCAAACGCCTTCAACAACAGCATTATTCCAGAAGACACCTTCTTCCCCAG<br>CCCAGGTAAGGGCAGCTTTGGTGCCTTCGCAGGCTGTTTCCTTGCTTCAGGAATGGCCA

Supplementary Table 6

Name (Locus_application)	Forward primer	Reverse primer
UROS_NGS	ACACTCTTTCCCTACACGACGCTCTTCCGATCT GGAATTTAGTCTCCCAGCAG	GTGACTGGAGTTCAGACGTGTGCTCTTCCGATCT CTTTTTGGTGTGCAGCTTT
TEX45_NGS	ACACTCTTTCCCTACACGACGCTCTTCCGATCT GAGCTGCTGCAAGCGCAG	GTGACTGGAGTTCAGACGTGTGCTCTTCCGATCT CAGCTCGGGCCAGCCGTA
PPP1R12C_site 1_NGS	ACACTCTTTCCCTACACGACGCTCTTCCGATCT GGGACCACCTTATATTCCCAGG	GTGACTGGAGTTCAGACGTGTGCTCTTCCGATCT ATCCTCTCTGGCTCCATCGT
PPP1R12C_site 2_NGS	ACACTCTTTCCCTACACGACGCTCTTCCGATCT ACCCCACAGTGGGGCCACTA	GTGACTGGAGTTCAGACGTGTGCTCTTCCGATCT GGAGAGAGATGGCTCCAGGAA
PLCB4_NGS	ACACTCTTTCCCTACACGACGCTCTTCCGATCT GGAACAGAAACCTGTTGGTGCC	GTGACTGGAGTTCAGACGTGTGCTCTTCCGATCT GGAAGAAAGTGGAGGGGTGAGA
ANK1_NGS	CTTTCCCTACACGACGCTCTTCCGATCT TCCCACTCCTCCCATGTGTT	GGAGTTCAGACGTGTGCTCTTCCGATCT CTTCGGCTCCACTTCCCTGC
SLX4_NGS	CTTTCCCTACACGACGCTCTTCCGATCT TCTGAGGGTGAGCTTGTCTTTG	GGAGTTCAGACGTGTGCTCTTCCGATCT TCTGGCAGAAGAACAAACCCT
DACT2_NGS	CTTTCCCTACACGACGCTCTTCCGATCT TCCCCGTCTTGACATGGCTG	GGAGTTCAGACGTGTGCTCTTCCGATCT GTGCTCACAAAGCAAGCACA
ZEB1_NGS	CTTTCCCTACACGACGCTCTTCCGATCT TGATAGCACTTGTCTTCTGTGTG	GGAGTTCAGACGTGTGCTCTTCCGATCT CTTTAAAGGTGGCTGACTAGGAG
GOLGA3_NGS	CTTTCCCTACACGACGCTCTTCCGATCT TCAGGCAGTGTTTCTGATAGGA	GGAGTTCAGACGTGTGCTCTTCCGATCT AAAGACCTCAGGGAACTAGAAC
MFGE8_NGS	CTTTCCCTACACGACGCTCTTCCGATCT GCCCTCTGGGAGACACTAAG	GGAGTTCAGACGTGTGCTCTTCCGATCT CCAGCTACACATCCCAGCTA
FAM134B_NGS	CTTTCCCTACACGACGCTCTTCCGATCT CTAGCTCAGCTGGAGTGGGC	GGAGTTCAGACGTGTGCTCTTCCGATCT GCCATGGGTAAATCAAGTCAGG
RP11-280G9.1_NGS	CTTTCCCTACACGACGCTCTTCCGATCT CCCTACCTATCTTACGGTTGGA	GGAGTTCAGACGTGTGCTCTTCCGATCT GAGCAGCAGATTTTTCTGGTGA
RASAL2_NGS	CTTTCCCTACACGACGCTCTTCCGATCT GGCAGCCCATCAAGAAGTAAT	GGAGTTCAGACGTGTGCTCTTCCGATCT AGTGATAACTTCTGTATCATAGTCTAGC
PAQR5_NGS	CTTTCCCTACACGACGCTCTTCCGATCT CAAAACCCCATTCCTGGCTG	GGAGTTCAGACGTGTGCTCTTCCGATCT GGTTTTCATGGGGAGGAAGAAG
KCNQ4_NGS	CTTTCCCTACACGACGCTCTTCCGATCT AACCCTGAAATGCATGTGTTGA	GGAGTTCAGACGTGTGCTCTTCCGATCT TCTGAAGAGATGGAGGAGGTATG
ABCA3_NGS	CTTTCCCTACACGACGCTCTTCCGATCT AGGGTGCGCCTACCTCATC	GGAGTTCAGACGTGTGCTCTTCCGATCT GTGGGGAGCATCTCGCCA
LARGE_NGS	CTTTCCCTACACGACGCTCTTCCGATCT GAATGAAGGGACTGCATATGGG	GGAGTTCAGACGTGTGCTCTTCCGATCT GTCCCAGACTTCTAGCTTCTAG
UROS_Sanger	TCCCAAGGCAGAGTCTGTGACC	CCTCTGTGCTGCAGTGCACTT
TEX45_Sanger	CACCGGGACTTTGCCTAC	TGTGAAGCAAATCGCCCA
PPP1R12C_Sanger	GACAACCCCAAAGTACCCCG	ATCCTCTCTGGCTCCATCGT
PLCB4_Sanger	AGTTACTGGCTGTCTTGCCAT	TTCTGAATCAAATGCCAATGCC
ANK1_Sanger	TCAACCCCAGCTCCATTGCAT	GTGTGGACTCACTGAGAATGCT
SLX4_Sanger	GAGTGCTGATTAGTGCCCAGT	GGGTAGGTTGACAACAAAGCTG
DACT2_Sanger	ATTCATAAGGGTTAGGTTCCCCA	ATGCCTGAATTCCAAGGCTATC
ZEB1_Sanger	CAGTCTTGAGCAGCCTAGCC	GGGGATGGTGTACTACTTCTGG
GOLGA3_Sanger	TGTGAGATTGATGCTACGGGTC	GACCCTCTAACACATTTCCCTC
MFGE8_Sanger	GGATAGACACTTGTGGAGTGAGG	GACATTAAGCATCATGCTCAAGTCA
FAM134B_Sanger	AGTCTCAGGTATTTCTTTAGGGC	AGCAGCAGGGAGTTTGTATTCT
RP11-280G9.1_Sanger	GGAAGTGTTGGTATTTAGGCAG	CCAACTAGAGTCTTGCTTTGCA
RASAL2_Sanger	GAAGTGTGCTAAGGGCACTG	CCCTTATGAGCAAAGCCAACA
PAQR5_Sanger	GGCTGGAGGAAAATCTGCAATGA	ATTTCTCAAGTCAAATGCCCCTG
KCNQ4_Sanger	TCAGGGAAACTGCTGGTGGC	GGACAGGAAGGCATGTAGGT
ABCA3_Sanger	CATGGAGGGGACATTGTTACAG	GGCTCACAACTCGACAGCC
LARGE_Sanger	CCAAAGCCAATGTCATCAGAGT	ATAGGACTGGTGCCCTCATGT
HIST1H2BJ_Sanger & donor	AAGGTTCTGAAGCAGGTCCAC	CGGGCAAATTCAAACTGACAAC
FBL_Sanger & donor	GAAAAACAGCCCAAAGCCCTGT	AGCAAAATGGCGACCACAACAA
RAB11A_Sanger & donor	GGTAGCTAGGAGTTCCAGGAC	ACGATGTGGGAGAAGGCAGTC
AAVS1_Donor	TGCTTTCTCTGACCAGCATT	GAGCAGAGCCAGGAACCCC
TRAC_Donor	TTTCAGGTTTCCTTGAGTGGCA	TGGCCATTCCTGAAGCAAGGAA
TRAC_Sanger	TTGTGCCTGTCCCTGAGTCC	TGGGCTGGGGAAGAAGGTGT
UROS_PacBio	TGACGTCAGATAAAGGATAAGGG	CCTTGACTCCTTACCTAGAGAAG
PLCB4_PacBio	GACCTAAGTGGTAGGCTGTGAT	AGCTTTGGGAGCATGAGAGAC
TRAC_PacBio	GGCACATGCAAAGTAGCCTAAG	CGGCCACTTTCAGGAGGAGG
p21_qPCR	AGGTGGACCTGGAGACTCTCAG	TCCTCTTGGAGAAGATCAGCCG
ActB_qPCR	GGGTCAGAAGGATTCCTATG	GGTCTCAAACATGATCTGGG

Supplementary Note 1

Extended discussion of REtarget scores

We introduced the REtarget score primarily as a criterion to select the most promising gRNAs during the recursive gRNA search process. The REtarget score incorporates information about the percentage of editing outcomes that an individual gRNA can retarget (cutting score) and about the percentage of resulting indels that are again suitable for retargeting (editing score; *e.g.,* predicted frequency of indel above user-defined threshold):

$$REtarget\ score = \frac{cutting\ score}{100} \times \frac{editing\ score}{100}$$

REtarget scores for individual gRNAs can take a value between 0 and 1. REtarget scores for individual gRNAs can be aggregated for all gRNAs belonging to a single level (level REtarget score) or all levels (total REtarget score):

$$level\ REtarget\ score(l) = \sum_{gRNAs\ g \in l} REtarget\ score(g)$$

$$total\ REtarget\ score = \sum_{l} {w_l}^{(l-1)} \times level\ REtarget\ score(l)$$

g denotes an individual gRNA, l an individual level, and w_l a weight factor that adjusts the influence of higher levels on the total REtarget score. Owed to the aggregation method, the upper bound of total REtarget scores equals the number of levels considered for the recursive search.

The REtarget score does not consider on- or off-target efficiencies of individual gRNAs or the epigenetic state of the targeted site, which are all factors contributing to the overall success of Recursive Editing. Apart from that, REtarget Scores depend on the sequence context, indel prediction tool, and search parameters. Distinct search parameters can result in different REtarget scores for the same gRNA. Thus, total and level REtarget scores themselves should not serve as a standalone selection criterion when comparing different genomic sites and their potential for Recursive Editing. The purpose of total and level REtarget scores is to inform researchers about the general suitability of given sites for Recursive Editing. Besides, the scores allow researchers to disregard sites with low scores, which are unlikely to yield substantial HDR improvements due to the low abundance of retargetable indels.

Theory underlying Recursive Editing

In mammalian cells, HDR frequencies are often low relative to indel formation, limiting its applicability or requiring subsequent enrichment steps. Recursive Editing represents a strategy to reduce these obstacles by enhancing overall HDR rates while simultaneously decreasing the overall percentage of undesired indels. Recursive Editing takes advantage of the observation that NHEJ and alt-EJ editing outcomes behave semi-deterministically and can be predicted *in silico* by computational tools such as inDelphi (https://indelphi.giffordlab.mit.edu/single)[1], FORECasT (https://partslab.sanger.ac.uk/)[2], and Lindel (https://lindel.gs.washington.edu/Lindel/)[3]. By harnessing the predictions of these tools, we can design gRNAs retargeting abundant indels for another round of genome editing. Retargeting creates the repeated opportunity for HDR to occur

in the presence of a donor template. The newly formed HDR products are additive to previous HDR outcomes, thereby enhancing overall HDR rates. The approach can be applied recursively as long as editing outcomes allow efficient retargeting. REtarget is a computational tool that incorporates this optimization scheme to find loci suitable for recursive editing and designs gRNAs that can be used in the laboratory to improve HDR.

Algorithmic scheme of REtarget to predicts suitable gRNAs for Recursive Editing

In order to identify genomic regions amenable to Recursive Editing, we developed a software tool for the automated design of multiple recursive gRNAs called REtarget (online version: https://recursive-editing.herokuapp.com. REtarget takes the sequence context around the genomic region or position of interest as input and utilizes Lindel or inDelphi to predict indel signatures of individual editing events (**Fig. 1b**). The first-level "initiating" gRNA can be either designed as part of the REtarget search or supplied by the user. Positions, where initial Editing is predicted to yield a small number of highly abundant indels, are considered for retargeting. These abundant indels serve as entry points for further gRNA sampling. During this subsequent optimization, REtarget samples NGG-PAM motifs proximal to the predicted outcomes of the prior round. REtarget designs the corresponding candidate gRNAs (step 1) and subsequently filters out ones with low predicted efficiencies (step 2). For each gRNA, REtarget calculates a score (REtarget score). This score incorporates both the abundance of the newly targeted indels as well as the fraction of editing outcomes suitable for retargeting (step 3-5). REtarget then selects candidate gRNAs with the highest REtarget scores for each "level" of Editing (step 5). Finally, the stopping criteria are checked (step 6) and the gRNA search is recursively continued if criteria are not satisfied. The search is stopped after a user-defined maximum number of targeting levels, if the REtarget scores of all gRNAs from a single level fall below a user-defined threshold, or if the global optimum is reached (*e.g.* there are no more paths forward for retargeting). The general suitability of a genomic site for Recursive Editing can be estimated by the total REtarget score, which is calculated as the sum of each level's REtarget score and has the total number of levels as the upper boundary.

REtarget comprises two distinct search modes: (i) REtarget can check specific sites (fixed position of first DSB) for their suitability for Recursive Editing and design gRNAs for this particular site. (ii) REtarget can search sequence windows (online version: up to 100 bp, local version: no limitation) for suitable loci that enable efficient HDR improvement. REtarget will list corresponding gRNAs for all loci of interest in the sequence window search mode. The local implementation of REtarget can be run with any genome of interest.

gRNA filtering criteria of REtarget

REtarget filters gRNAs based on both their on-target and off-target efficiency. REtarget utilizes Doench 2014 scores to assess the on-target efficiency of gRNAs[4]. The corresponding script *doench_score.py* was implemented by the authors of CRISPOR[5]. The local version of REtarget utilizes FlashFry[6] to predict genome-wide off-targets with up to four mismatches and removes gRNAs with more off-targets than a user-defined threshold. Additionally, gRNAs can be filtered according to their MIT specificity score[7] and Doench CFD off-target specificity score[8].

It is important to note that the online version of REtarget does not perform a genome-wide search for gRNA off-targets to keep processing time in a reasonable range. Only gRNAs targeting recognition sites proximal to the initial DSB (±50 bp) in the WT sequence are automatically disregarded. Due to this relaxed filtering, it is crucial to check promising REtarget outputs for the possibility of genome-wide off-targets. To enable this, REtarget results can be automatically run in popular global off-target prediction tools such as CRISPOR[5] and CasOFFinder[9]. For instance, by clicking the corresponding lettering, users will be forwarded to the CRISPOR web server results page, showing off-target efficiency scores and the number of genome-wide off-targets with up to four mismatches for all REtarget gRNAs.

To ensure the additive accumulation of HDR outcomes, the donor template should be designed such that HDR alleles and the template itself are not themselves targeted. This can be accomplished by standard mutation of PAMs or seed sequences in the template. HDR template retargeting is often implicitly prevented by automatic changes in the seed region of gRNAs that target downstream indel alleles since bulges are particularly refractory to Cas9 activity. Users can specify their HDR template of interest, and REtarget will disregard all gRNAs for the recursive search process that would cut the donor template (no mismatches and NGG-PAM).

Furthermore, users can integrate previously generated experimental information in the recursive search process by specifying a blacklist of gRNAs that should be disregarded under any circumstances. This feature is of interest if a gRNA has experimentally proven insufficient, *e.g.,* due to limited cutting efficiency. Apart from the criteria mentioned above, gRNAs can be filtered according to their GC-content, the occurrence of homopolymer-, and TTT-stretches. REtarget does not consider the epigenetic state of the targeted DNA for gRNA generation and selection.

<u>REtarget syntax to describe Recursive Editing and associated gRNAs</u>

In the main text and in the online version of REtarget, we use the following syntax to describe Recursive Editing and its associated gRNAs: each "level" of Editing is denoted as an uppercase letter in the order of the alphabet (*i.e.* A denotes 1st level, B denotes 2nd level, etc.). Here, a level refers to a given set of indel outcomes, which can be targeted with either sequential or simultaneous delivery of editing reagents. Each gRNA at a given level is labeled with a number (*i.e.* 1, 2, etc.) corresponding to the predicted ranked abundance from REtarget. For instance, a 2nd level gRNA targeting the 2nd most abundant predicted outcome of this level is referred to as gRNA B2 (**Fig. 1c**).

The local version of REtarget uses the syntax that is utilized during the recursive gRNA optimization process. Each gRNA targeting a given sequence is labeled with a number (*i.e.* 1, 2, etc.) corresponding to the predicted ranked abundance from REtarget. gRNA labels for each level are concatenated to each other, separated by the character "_" (*i.e.* 1_1 denotes the gRNA of the 2nd level that targets the most abundant indel introduced by the 1st level gRNA 1). This annotation allows us to preserve information about the origin of indels in the Recursive Editing scheme. The gRNA targeting the WT genotype is always annotated as 1.

Usage of REtarget web tool

A user guide for the web tool is available online (https://recursive-editing.herokuapp.com). In summary, the online version of REtarget requires the following user inputs (**Supplementary Fig. 8**):

1. Sequence region of interest (number 1 in **Supplementary Fig. 8**): users can copy-paste a sequence region of interest, between 100 bp and 1,000 bp in length, into the dedicated field.
2. Search mode (number 2 in **Supplementary Fig. 8**): users can specify if they want to check a specific locus or search a sequence window for loci suitability for recursive Editing. (i) To start the search from a specific position, users may either indicate the first gRNA or the first cut site to be used for the search. REtarget will check the input and autofill the complementary field once the user presses enter or leaves the input field with his or her cursor. Note that REtarget only accepts NGG-PAMs. If the search is started before those processes are completed, REtarget will raise an error. The cut position is defined relative to the start of the selected sequence (the first bp in the copy-pasted sequence/selected chromosome has position 0). (ii) To search a sequence window, users must specify the start and end position of the region to be searched. Positions are defined relative to the start of the selected sequence (first bp = position 0). REtarget will evaluate the suitability of all NGG-PAM editing sites within the selected sequence window.
3. HDR template (not required; number 3 in **Supplementary Fig. 8**): users can specify their HDR template to disregard gRNAs targeting the template.
4. Tool to predict indel signatures (number 4 in **Supplementary Fig. 8**): users can decide if Lindel or inDelphi should be utilized to predict indel signatures for single editing events. Following previous studies and our own experimental results, we recommend using Lindel[3,10]. inDelphi additionally allows users to select the cell type of interest. For cell types without major defects in DNA repair, the authors recommend selecting mESC[1].
5. Search settings (number 5 in **Supplementary Fig. 8**): users can state if the search should be conducted with default settings or adjust all parameters according to their particular use case (*cf.* **Supplementary Note 1**).
6. REtarget will start the HDR optimization search and output results once the “Start search” button (number 6 in **Supplementary Fig. 8**) is clicked.

Explanation of REtarget search parameters

Users can adjust the following parameters (variable names used in the python implementation referenced in brackets):

1. Maximal number of gRNAs (MAX_GUIDE_NUM): the maximal number of gRNAs that REtarget will generate as output. Once the maximal number of gRNAs is reached, REtarget will compare the REtarget scores of newly designed and previously selected gRNAs and keep those gRNAs resulting in the highest total REtarget score. Default: 5
2. Maximal number of levels (MAX_LEVEL_NUM): gRNAs in the same level require the same number of editing rounds for their target genotype to be generated. Level 1 comprises, by definition, only the first gRNA targeting the WT sequence (= 1st round of Editing). All editing outcomes resulting from gRNA1 give rise to potential level 2 gRNAs (= 2nd round of Editing). The maximal number of levels serves as a stopping criterion. REtarget will terminate after MAX_LEVEL_NUM levels if the optimization process has not been stopped before due to other criteria. Default: 3
3. Number of predicted editing outcomes considered per gRNA (MAX_GUIDES): Specifies how many predicted indels resulting from a single editing event are considered in the optimization process. The value should be lower or equal to the maximal number of gRNAs. Default: 3
4. Number of NGG-PAMs considered to find new candidate gRNAs (MAX_PAM_NUM): Each sufficiently abundant editing outcome will serve as a seed sequence for the generation of new candidate gRNAs. For this, a particular sequence window around the previous cut site will be searched for forward and reverse NGG-PAMs. This parameter specifies how many of the corresponding gRNAs should be further evaluated. Before this filter is applied, corresponding gRNAs are sorted according to their on-target cutting efficiency. Thus, only the most promising gRNAs are handed over to the next step. Default: 5
5. Minimal frequency of editing outcomes (SINGLE_FREQ_CUT): All editing outcomes with lower predicted frequencies will be disregarded. Note that meaningful cutoffs can be markedly different depending on the prediction tool utilized. Default: 5%
6. Minimal level REtarget score (LEVEL_FREQ_CUT): This parameter serves as the primary stopping criterion for the algorithm. Once the level REtarget score falls below this user-defined threshold, the optimization process will terminate and output the results. Note that REtarget scores for higher layers are usually very small. Thus, setting a high threshold may unintentionally result in early termination of the optimization process. Default: 0.05
7. Factor to down weight cutting scores of higher levels (LEVEL_WEIGHT_FACTOR): A factor smaller than 1 will downweigh the cutting scores of higher levels according to the following formula:

   $$cutting\ score\ new\ =\ cutting\ score\ old\ \times\ LEVEL\_WEIGHT\_FACTOR^{(l-1)}$$

   l denotes the corresponding level number. Default: 1 (no down weighting)
8. Factor to adjust REtarget scores of higher layers (LEVEL_SCORE_FACTOR): A factor smaller than 1 will down weight the Level REtarget scores of higher levels according to the formula:

$$level\ REtarget\ score(l) = \sum_{gRNAs\ g \in l} REtarget\ score(g)$$

l denotes the corresponding level number, g individual gRNAs. Users should not use values < 1 for LEVEL_WEIGHT_FACTOR and LEVEL_SCORE_FACTOR at the same time. Default: 1 (no down weighting)

9. Model used to predict editing outcomes (MODEL): 0 = Lindel (default), 1 = inDelphi
10. Cell type to initialize inDelphi (CELLTYPE_MODEL): value will be disregarded if Lindel is selected as a model. Default: "mESC"
11. Minimal overlap of candidate gRNAs with the right sequence arm of the previous cut site (MIN_OVERLAP): Ensures that gRNAs target unique sequences that are generated in the process of Recursive Editing, not lower-level editing outcomes or the WT-sequence. A value of 4, for instance, specifies that there is an overlap of at least 3 bp between the candidate gRNA and the right sequence arm of the previous cut site. Accordingly, a value of -1 means that the sequence "GG" of a potential PAM is centered around the previous cut site (corresponding to an overlap of -2 between candidate gRNA and the right sequence arm of the previous cut site. Specificity due to novel PAM-generation). Default: -1
12. Maximal overlap of candidate gRNAs with the right sequence arm of the previous cut site (MAX_OVERLAP): A value of 10, for instance, means that no candidate gRNA would be selected with an overlap between candidate gRNA and the right sequence arm of the previous cut site larger than 10 bp. Default: 10
13. Minimal Level REtarget Score of Level 1 (L1_CUT). The search will terminate without returning results if the criterion is not met. Default: 0.4
14. Minimal Level REtarget Score of Level 2 (L2_CUT). The search will terminate without returning results if the criterion is not met. Default: 0.2
15. Minimal number of Levels (L_MIN_SAVE): Results will not be stored if the number of resulting levels is smaller than this parameter. Not relevant for the online version of REtarget. Default: 2
16. Check for endogenous recognition sites in proximity to the initial cut site (CHECK_OFFT_PROX): Defines if gRNAs in levels > 1 targeting the WT sequence in proximity to the initial cut site should be sorted out. gRNAs that target lower-level editing outcomes but not the WT sequence will not be sorted out as this would also reduce undesired editing outcomes and potentially increase HDR. Default: True
17. Check for genome-wide off-targets (CHECK_OFFT_GENOME): defines if FlashFry should be used to search for off-targets (up to 4 mismatches). Not relevant for the online version of REtarget. Default: True. The name of the previously generated FlashFry database can be specified using the parameter FF_DATABASE_NAME, the path to the required java executable using JDK_PATH. FlashFry will be started utilizing the taskset command if USE_TASKSET_JKD is set to True (default).
18. Maximal number of off-targets with 0-4 mismatches (MAX_OFF_TAR): threshold for off-target based gRNA filtering. Not relevant for the online version of REtarget. Default: [0, 2, 20, 200, 2000]

19. Filter gRNAs based on off-target scores (CHECK_OFFT_SCORES). Not relevant for the online version of REtarget. Default: False
20. Minimal Doench 2014 on-target efficiency score (D2014ON_MIN): gRNAs with a Doench score lower than this threshold will be disregarded. Default: 0.05
21. Maximal Doench CFD score for gRNA with highest off-target activity (DCFD_MAXOT_MAX): gRNAs with a score higher than this threshold will be disregarded. Default: 0.75
22. Minimal Doench CFD off-target specificity score (DCFD_SPEC_MIN): gRNAs with a Doench CFD score lower than this threshold will be disregarded. Default: 0.5
23. Minimal MIT specificity score (HSU2013_MIN): gRNAs with an MIT specificity score lower than this threshold will be disregarded. Default: 0.5
24. Filter gRNAs based on GC-content (CHECK_GC): Default: False
25. Minimal GC-content of gRNA (GC_LOW): gRNAs with a GC-content lower than this threshold will be disregarded. Default: 0.1
26. Maximal GC-content of gRNA (GC_HIGH): gRNAs with a GC-content higher than this threshold will be disregarded. Default: 0.9
27. Filter gRNAs based on homopolymers (CHECK_POLY_N). Default: False
28. Check if gRNAs target given HDR template (CHECK_TEMPLATE). Default: False (online version: True if template specified). HDR template can be defined using the parameter HDR_TEMPLATE.
29. Filter gRNAs based user-defined blacklist (CHECK_BLACKLIST). Default: False. gRNA blacklist can be defined using the parameter BLACKLIST.
30. Specify if gRNAs of final level should be designed according to recursive optimization scheme (highest REtarget score) or based on their on-target efficiency (Doench 2014 score, FINAL_BY_D2014ON): Default: False (final layer not based on Doench 2014 score); Online version default: True
31. Logging mode (VERBOSE): Default: True

<u>Explanation of REtarget result files</u>

REtarget generates three output files if a site fulfills the criteria defined by the aforementioned parameters:

1. res_df.csv: will be generated for each search and contains a single entry for each genomic site amenable to Recursive Editing. The columns contain the following information for each site:
   Id (identifier for the genomic site in the format id_[position relative to start position of input sequence]_[orientation of gRNA in relation of input sequence]), Number of levels, Number of guides, Guide RNAs (list of all gRNAs), Total REtarget score, REtarget score level 1, REtarget score level 2.
2. guide_df.csv: will be generated for each site listed in res_df.csv and contains a single entry for each gRNA involved in Recursive Editing. The columns contain the following information for each gRNA:
   Guide_ID, Guide_RNA, Level, REtarget_Score, MGA_REtarget_Score (accumulated REtarget scores if single gRNA appears multiple times), Cutting_Score, Editing_Score,

Doench2014_Score, DoenchCFD_MaxOT (OT: off-target), DoenchCFD_SpecScore (off-target score), Hsu2013_Score, Number_Off_Targets, Mismatches_Closest_OT, Number_Closest_OT, Guide_Orientation, PAM, Target, Target_Type, Cut_Position, Target_Origin, Multi (specifies if gRNA appears multiple times).

3. edit_df.csv: will be generated for each site listed in res_df.csv and contains all predicted editing outcomes for each gRNA. The columns contain the following information for each retargetable indel:
   Category (insertion or deletion), Genotype position (start position of indel in relation to cut site), Inserted Bases, Length (length of indel), Predicted frequency (relative indel frequency in %), Genotype, REtarget_Score_Contribution, Seq_Type (orientation of genotype in relation to input sequence), Cutsite, Edit_ID, Guide_ID, Level.
4. Users can also download a file containing the parameters applied for the search. Parameters are listed comma-separated in the following order:
   MAX_GUIDE_NUM, MAX_LEVEL_NUM, MAX_GUIDES, MAX_PAM_NUM, SINGLE_FREQ_CUT, LEVEL_FREQ_CUT, LEVEL_WEIGHT_FACTOR, LEVEL_SCORE_FACTOR, MIN_DSCORE, GC_LOW, GC_HIGH, BLACKLIST, MIN_OVERLAP, MAX_OVERLAP, L1_CUT, L2_CUT, MODEL, CELLTYPE_MODEL, CHECK_OFFT_PROX, CHECK_GC, CHECK_POLY_N, CHECK_TTT, CHECK_TEMPLATE, CHECK_BLACKLIST, FINAL_BY_D2014ON

Genome-wide search for loci amenable to Recursive Editing

We used REtarget to search the human genome (GRCh38, Genbank) for sites amenable for Recursive Editing. Results from our genome-wide search are reported as **Supplementary Data 1**. This table specifies initial target sequences and genomic positions of all gRNA hits from the genome-wide search. For exact data on corresponding higher-level gRNAs, please consult the online or local implementation of REtarget and use the specified first-level gRNA as an input guide. The search was conducted with relatively strict search parameters to restrict the computational complexity. Thus, genomic sites not present in the dataset will be suitable for Recursive Editing, as well. Note that the database was initially generated without using FlashFry for off-target prediction to limit the computational complexity. All gRNAs were subsequently compiled and filtered according to their off-targets, yielding the final database.

Search parameters applied for genome-wide search:

- MAX_GUIDE_NUM = 10
- MAX_LEVEL_NUM = 3
- MAX_GUIDES = 3
- MAX_PAM_NUM = 10
- SINGLE_FREQ_CUT = 10
- LEVEL_FREQ_CUT = 0.1
- LEVEL_WEIGHT_FACTOR = 1
- LEVEL_SCORE_FACTOR = 1
- MODEL = 0
- CELLTYPE_MODEL = 'mESC'
- MIN_OVERLAP = -1
- MAX_OVERLAP = 10
- L1_CUT = 0.6
- L2_CUT = 0.3
- L_MIN_SAVE = 2
- CHECK_OFFT_GENOME = True
- CHECK_OFFT_PROX = True
- MAX_OFF_TAR = [0, 2, 20, 200, 2000]
- CHECK_OFFT_SCORES = False
- D2014ON_MIN = 0.25
- DCFD_MAXOT_MAX = 0.75
- DCFD_SPEC_MIN = 0.5
- HSU2013_MIN = 0.5
- CHECK_GC = True
- GC_LOW = 0.1
- GC_HIGH = 0.9
- CHECK_POLY_N = False
- CHECK_TTT = False

FINAL_BY_D2014ON = False

Genome-wide start and stop codon search for loci amenable to Recursive Editing

We used REtarget to search all start and stop codons of the human genome extracted from RefSeq (version as of 12/2021) for sites amenable for Recursive Editing. Results from our genome-wide search are reported in **Supplementary Data 2** (only initial target sequences and genomic positions). For exact data on corresponding higher-level gRNAs, please consult the online or local implementation of REtarget and use the specified first-level gRNA as an input guide. The database was initially generated without using FlashFry for off-target prediction to limit the computational complexity. All gRNAs were subsequently compiled and filtered according to their off-targets, yielding the final database.

Search parameters applied for database generation:

- MAX_GUIDE_NUM = 5
- MAX_LEVEL_NUM = 3
- MAX_GUIDES = 3
- MAX_PAM_NUM = 10
- SINGLE_FREQ_CUT = 10
- LEVEL_FREQ_CUT = 0.1
- LEVEL_WEIGHT_FACTOR = 1
- LEVEL_SCORE_FACTOR = 1
- MODEL = 0
- CELLTYPE_MODEL = 'mESC'
- MIN_OVERLAP = -1
- MAX_OVERLAP = 10
- L1_CUT = 0.35
- L2_CUT = 0.15
- L_MIN_SAVE = 2
- CHECK_OFFT_GENOME = True
- CHECK_OFFT_PROX = True
- MAX_OFF_TAR = [0, 2, 20, 200, 2000]
- CHECK_OFFT_SCORES = False
- D2014ON_MIN = 0.05
- DCFD_MAXOT_MAX = 0.75
- DCFD_SPEC_MIN = 0.5
- HSU2013_MIN = 0.5
- CHECK_GC = False
- GC_LOW = 0.1
- GC_HIGH = 0.9
- CHECK_POLY_N = False
- CHECK_TTT = False
- FINAL_BY_D2014ON = False

Search of ClinVar database for loci amenable to Recursive Editing

We used REtarget to find the best Recursive Editing gRNA set for each of the 94,000+ annotated pathogenic mutations in ClinVar (version as of 11/2021), excluding indels >50 bp that are less ideal for ssODN donors and applying looser parameters than in the previous genome-wide search for globally optimal reagents. Results are reported as **Supplementary Data 3** (only initial target sequences and genomic positions). For exact data on corresponding higher-level gRNAs, please consult the online or local implementation of REtarget and use the specified first-level gRNA as an input guide. The database was initially generated without using FlashFry for off-target prediction to limit the computational complexity. All gRNAs were subsequently compiled and filtered according to their off-targets, yielding the final database.

Search parameters applied for database generation:

- MAX_GUIDE_NUM = 5
- MAX_LEVEL_NUM = 3
- MAX_GUIDES = 3
- MAX_PAM_NUM = 10
- SINGLE_FREQ_CUT = 10
- LEVEL_FREQ_CUT = 0.1
- LEVEL_WEIGHT_FACTOR = 1
- LEVEL_SCORE_FACTOR = 1
- MODEL = 0
- CELLTYPE_MODEL = 'mESC'
- MIN_OVERLAP = -1
- MAX_OVERLAP = 10
- L1_CUT = 0.35
- L2_CUT = 0.15
- L_MIN_SAVE = 2
- CHECK_OFFT_GENOME = True
- CHECK_OFFT_PROX = True
- MAX_OFF_TAR = [0, 2, 20, 200, 2000]
- CHECK_OFFT_SCORES = False
- D2014ON_MIN = 0.05
- DCFD_MAXOT_MAX = 0.75
- DCFD_SPEC_MIN = 0.5
- HSU2013_MIN = 0.5
- CHECK_GC = False
- GC_LOW = 0.1
- GC_HIGH = 0.9
- CHECK_POLY_N = False
- CHECK_TTT = False
- FINAL_BY_D2014ON = False

Supplementary References

1. Shen, M. W. *et al.* Predictable and precise template-free CRISPR editing of pathogenic variants. *Nature* **563**, 646–651 (2018).
2. Allen, F. *et al.* Predicting the mutations generated by repair of Cas9-induced double-strand breaks. *Nat. Biotechnol.* **37**, 64–72 (2019).
3. Chen, W. *et al.* Massively parallel profiling and predictive modeling of the outcomes of CRISPR/Cas9-mediated double-strand break repair. *Nucleic Acids Res.* **47**, 7989–8003 (2019).
4. Doench, J. G. *et al.* Rational design of highly active sgRNAs for CRISPR-Cas9–mediated gene inactivation. *Nat. Biotechnol.* **32**, 1262–1267 (2014).
5. Concordet, J.-P. & Haeussler, M. CRISPOR: intuitive guide selection for CRISPR/Cas9 genome editing experiments and screens. *Nucleic Acids Res.* **46**, W242–W245 (2018).
6. McKenna, A. & Shendure, J. FlashFry: a fast and flexible tool for large-scale CRISPR target design. *BMC Biol.* **16**, 74 (2018).
7. Hsu, P. D. *et al.* DNA targeting specificity of RNA-guided Cas9 nucleases. *Nat. Biotechnol.* **31**, 827–832 (2013).
8. Doench, J. G. *et al.* Optimized sgRNA design to maximize activity and minimize off-target effects of CRISPR-Cas9. *Nat. Biotechnol.* **34**, 184–191 (2016).
9. Bae, S., Park, J. & Kim, J.-S. Cas-OFFinder: a fast and versatile algorithm that searches for potential off-target sites of Cas9 RNA-guided endonucleases. *Bioinformatics* **30**, 1473–1475 (2014).
10. Tatiossian, K. J. *et al.* Rational Selection of CRISPR-Cas9 Guide RNAs for Homology-Directed Genome Editing. *Mol. Ther.* **29**, 1057–1069 (2021).
