# Supplementary material for: Recursive Editing improves homology-directed repair through retargeting of undesired outcomes
Source: Nat Commun. 2022 Aug 5;13:4550. doi: 10.1038/s41467-022-31944-7 (PMC9356142; doi:10.1038/s41467-022-31944-7)
Supplement: Supplementary file 2 — Reporting Summary [file 41467_2022_31944_MOESM2_ESM.pdf]

nature research

Corresponding author(s): Eric J. Aird, Jacob E. Corn

Last updated by author(s): Jun 27, 2022

Reporting Summary

Nature Research wishes to improve the reproducibility of the work that we publish. This form provides structure for consistency and transparency in reporting. For further information on Nature Research policies, see our Editorial Policies and the Editorial Policy Checklist.

Statistics

For all statistical analyses, confirm that the following items are present in the figure legend, table legend, main text, or Methods section.

n/a	Confirmed	
☐	☒	The exact sample size (n) for each experimental group/condition, given as a discrete number and unit of measurement
☐	☒	A statement on whether measurements were taken from distinct samples or whether the same sample was measured repeatedly
☐	☒	The statistical test(s) used AND whether they are one- or two-sided<br>*Only common tests should be described solely by name; describe more complex techniques in the Methods section.*
☒	☐	A description of all covariates tested
☒	☐	A description of any assumptions or corrections, such as tests of normality and adjustment for multiple comparisons
☐	☒	A full description of the statistical parameters including central tendency (e.g. means) or other basic estimates (e.g. regression coefficient) AND variation (e.g. standard deviation) or associated estimates of uncertainty (e.g. confidence intervals)
☐	☒	For null hypothesis testing, the test statistic (e.g. F, t, r) with confidence intervals, effect sizes, degrees of freedom and P value noted<br>*Give P values as exact values whenever suitable.*
☒	☐	For Bayesian analysis, information on the choice of priors and Markov chain Monte Carlo settings
☒	☐	For hierarchical and complex designs, identification of the appropriate level for tests and full reporting of outcomes
☐	☒	Estimates of effect sizes (e.g. Cohen's d, Pearson's r), indicating how they were calculated

Our web collection on statistics for biologists contains articles on many of the points above.

Software and code

Policy information about availability of computer code

Data collection	The latest versions of inDelphi (v0.18.1) (Shen et al. 2018) and Lindel (version as of 05/2021) (Chen et al. 2019) were downloaded from GitHub and run locally to predict editing outcomes. We used a script from the CRISPOR web tool (v4.99) to calculate Doench Scores (Concordet & Haeussler 2018). Off-target scoring was performed using FlashFry (v1.11) (McKenna & Shendure. REtarget was implemented in Python (v3.7.10), tested on a Linux operating system, and requires the modules Biopython (v1.78), Kiwisolver (v1.3.1), Matplotlib (v3.2.2), Numpy (v1.15.3), Pandas (v0.23.4), Scikit-learn (v0.20.0) and Scipy (v1.1.0). The online version of REtarget was built with Dash (v2.0.0) and Plotly (v5.3.1). Flow cytometry: Attune Nxt Software (v3.2.1). REtarget code is freely available to academic users upon request and available for license from ETH. The web-based version of REtarget is free for use and can be accessed at https://recursive-editing.herokuapp.com/ .
Data analysis	Illumina amplicon sequencing: Crispresso2 (v2.0.20b) (Clement et al. 2019). PacBio amplicon sequencing: SMRT Link (v10.2.0.133434), genomcov (v2.27.1), and BBMAP v38.69. DISCOVER-Seq analysis: BLENDER (v1.0.1; https://github.com/staciawyman/blenvder). Flow cytometry: FlowJo (v10.8). All graphs were produced in Prism (v9.3.1; GraphPad), and figures were assembled using Affinity Designer (1.10.5; Serif). Bowtie2 (v2.4.5)

For manuscripts utilizing custom algorithms or software that are central to the research but not yet described in published literature, software must be made available to editors and reviewers. We strongly encourage code deposition in a community repository (e.g. GitHub). See the Nature Research guidelines for submitting code & software for further information.

Data

Policy information about availability of data

All manuscripts must include a data availability statement. This statement should provide the following information, where applicable:

- Accession codes, unique identifiers, or web links for publicly available datasets
- A list of figures that have associated raw data
- A description of any restrictions on data availability

GRCh38 was downloaded from GenBank. Sequencing data is deposited in SRA BioProject PRJNA837763 (https://www.ncbi.nlm.nih.gov/bioproject/PRJNA837763). Source Data are provided with this paper. The REtarget-generated databases are available as Supplementary Data 1-3.

Field-specific reporting

Please select the one below that is the best fit for your research. If you are not sure, read the appropriate sections before making your selection.

[x] Life sciences [ ] Behavioural & social sciences [ ] Ecological, evolutionary & environmental sciences

For a reference copy of the document with all sections, see nature.com/documents/nr-reporting-summary-flat.pdf

Life sciences study design

All studies must disclose on these points even when the disclosure is negative.

Sample size	No sample size calculations were performed. Sample size was determined to be adequate based on the consistency of measurable differences and previously published literature in the genome editing field (see Anzalone, 2019, Nature for example).
Data exclusions	No data were excluded from analysis expect in the case of failed experiments due to other factors (loss of reagent activity, contamination).
Replication	All experiments were performed with at least two biological replicates. Each data point represents an individual biological replicate as listed in figure captions. All replications were successful.
Randomization	No randomization was performed. All independent biological replicates were treated identically.
Blinding	No blinding was performed as data were not subjective. Cells were grown in identical conditions.

Reporting for specific materials, systems and methods

We require information from authors about some types of materials, experimental systems and methods used in many studies. Here, indicate whether each material, system or method listed is relevant to your study. If you are not sure if a list item applies to your research, read the appropriate section before selecting a response.

Materials & experimental systems

n/a	Involved in the study	
[ ]	[x]	Antibodies
[ ]	[x]	Eukaryotic cell lines
[x]	[ ]	Palaeontology and archaeology
[x]	[ ]	Animals and other organisms
[x]	[ ]	Human research participants
[x]	[ ]	Clinical data
[x]	[ ]	Dual use research of concern

Methods

n/a	Involved in the study	
[ ]	[x]	ChIP-seq
[ ]	[x]	Flow cytometry
[x]	[ ]	MRI-based neuroimaging

Antibodies

Antibodies used	Anti-MRE11 (Novus Biologicals NB100-142; polyclonal; lot U3) was used for the DISCOVER-Seq experiment.<br>Brilliant Violet 421 anti-human TCRα/β (BioLegend 306722; Clone IP26; lot B320285) was used for flow cytometry (1:100 dilution).<br>Alexa Fluor 647 anti-myc tag (Cell Signaling 2233; Clone 9B11; lot 25) was used for flow cytometry (1:50 dilution) to stain for BCMA-CAR.
Validation	Novus NB100-142: This antibody has been validated for ChIP via its usage in the DISCOVER-Seq publication (Wienert, et al 2019, Science). It has been cited 204 times according to the Novus Biologicals website (accessed 3 March 2022). Validated by the manufacturer using genetic perturbation of the target protein.<br><br>BioLegend 306722: Each lot of this antibody is quality control tested by immunofluorescent staining with flow cytometric analysis by the manufacturer. It has been cited 6 times according to the BioLegend website (accessed 7 April 2022). Validated by the

manufacturer using CRISPR-mediated knockout of the target protein.

Cell Signaling 2233: The monoclonal antibody is produced by immunizing animals with a synthetic peptide corresponding to residues 410-419 of human c-Myc (EQKLISEEDL). It has been cited 45 times according to the BioLegend website (accessed 7 April 2022). This antibody was validated for by the manufacturer using cells transfected with myc-tagged protein.

Eukaryotic cell lines

Policy information about cell lines

Cell line source(s)	Cell lines (HEK-293T, K-562, HCT-116, RPE1) were obtained from ATCC or Berkeley Cell Culture.
Authentication	STR profiling
Mycoplasma contamination	All cell lines were routinely tested for mycoplasma (MycoAlert; Lonza) and tested negative.
Commonly misidentified lines (See ICLAC register)	No commonly misidentified lines were used in this study.

ChIP-seq

Data deposition

☒ Confirm that both raw and final processed data have been deposited in a public database such as GEO.

☒ Confirm that you have deposited or provided access to graph files (e.g. BED files) for the called peaks.

Data access links *May remain private before publication.*	ChIP data is deposited in SRA BioProject PRJNA837763
Files in database submission	Files are included in the SRA submission.
Genome browser session (e.g. UCSC)	Not applicable.

Methodology

Replicates: One replicate per sample (UROS gA1, UROS gB1, UROS gC1, UROS gA1B1C1, non-targeting guide). Additional replicates are not required for this assay; however, a non-targeting control guide has to be present.

Sequencing depth: The target read depth was 20 million reads per sample on a 150 bp paired-end Illumina NextSeq2000. 96% of reads mapped uniquely.

Antibodies: Polyclonal antibody against MRE11. Catalog # NB100-142, Novus Biologicals, Lot U3

Peak calling parameters:

Samples: UROS gA1, UROS gB1, UROS gC1, UROS gABC
Control: non-targeting guide
Index file: UCSC hg38 Bowtie2 index file

Trim and align:

```
output=" " // specify output folder
READ1=" " // location and name of read1
READ2=" " // location and name of read2
name=" " // sample name

cutadapt \
-q 20 \
-m 20 \
-j 12 \
--times 4 \
-a TruSeq_R1=AGATCGGAAGAGCACACGTCTGAACTCCAGTCA \
-A TruSeq_R2=AGATCGGAAGAGCGTCGTGTAGGGAAAGAGTGT \
-o ${output}/${READ1}.trimmed \
-p ${output}/${READ2}.trimmed \
${READ1} \
${READ2} \
> ${output}/${i}_stderr_cutadapt_trimmed.log && \
\
bowtie2 \
--local \
--very-sensitive-local \
--threads 24 \
```

```
--phred33 \
--maxins 1000 \
-x ~/data/genomes/Homo_sapiens/UCSC/hg38/Sequence/Bowtie2Index/genome \
-1 ${output}/${READ1}.trimmed \
-2 ${output}/${READ2}.trimmed \
2> ${output}/${i}stderr_bowtie2.trimmed.log\
\
| samtools view -S -b - \
| samtools sort -@ 20 - -o ${output}/${name}.sorted.bam && \
\
samtools index ${output}/${name}.sorted.bam && \
\
rm ${output}/${READ1}.trimmed && \
rm ${output}/${READ2}.trimmed && \
bamCoverage -b ${output}/${name}.sorted.bam -o ${output}/${name}.coverage.bw

Blender:
sh run_blender.sh <path to reference genome> \
      <path to IP bamfile> \
      <path to control bamfile> \
      <guide sequence> <output directory> ["options"]
```

Data quality	BLENDER uses both IP and control samples, as well as filtering ENCODE blacklist locations in the genome. It does not use conventional ChIP-Seq peak calling
Software	Bowtie2 was used to align the reads to the human genome. Our custom software BLENDER (https://github.com/staciawyman/blender) was used for off-target peak calling.

Flow Cytometry

Plots

Confirm that:

- [x] The axis labels state the marker and fluorochrome used (e.g. CD4-FITC).
- [x] The axis scales are clearly visible. Include numbers along axes only for bottom left plot of group (a 'group' is an analysis of identical markers).
- [x] All plots are contour plots with outliers or pseudocolor plots.
- [x] A numerical value for number of cells or percentage (with statistics) is provided.

Methodology

Sample preparation	For K-562 staining of GFP, 5 to 7 days post-electroporation cells were pelleted, washed 1x with PBS, resuspended in a PBS / 10 % FBS solution, and analyzed immediately.<br>For cell surface antibody staining, 3 days post-electroporation cells were pelleted, resuspended in PBS / 2 % FBS solution containing antibody, and incubated 10-30 min at RT. Cells were then pelleted, washed once, and resuspended for analysis. The following antibodies were used: 1:100 dilution of Brilliant Violet 421 anti-human TCRα/β (Clone IP26; BioLegend 306722) and 1:50 of Alexa Fluor 647 anti-myc tag (Clone 9B11; Cell Signaling 2233).
Instrument	Attune NxT Flow Cytometer (with autosampler), Thermo Fisher
Software	Flow cytometry data was collected using Attune Nxt Software v3.2.1 and analyzed with FlowJo v10.8.
Cell population abundance	For each sample, 50,000 events (gated on single cells) were collected.
Gating strategy	Cells were first gated for live cells (SSC-A vs FSC-A), then gated for single cells (FSC-H vs FSC-A). GFP positive cells were then gated on SSC-A vs BL1-GFP, using a donor-only control to establish the gate. BCMA-CAR HDR was assessed by gating on Brilliant Violet 421 (TCR) versus Alexa Fluor 647 (myc-CAR), using a donor-only control to establish the gate.

- [x] Tick this box to confirm that a figure exemplifying the gating strategy is provided in the Supplementary Information.
