# Supplementary material for: Recursive Editing improves homology-directed repair through retargeting of undesired outcomes
Source: Nat Commun. 2022 Aug 5;13:4550. doi: 10.1038/s41467-022-31944-7 (PMC9356142; doi:10.1038/s41467-022-31944-7)
Supplement: Supplementary file 6 — Description of Additional Supplementary Files [file 41467_2022_31944_MOESM6_ESM.pdf]

Title: Supplementary Data 1.
Description: REtarget genome-wide search database

Title: Supplementary Data 2.
Description: REtarget start/stop codon search database

Title: Supplementary Data 3.
Description: REtarget pathogenic ClinVar search database
